# Protocol to implement and evaluate a culturally secure, strength-based, equine-assisted learning program, "Yawardani Jan-ga" (horses helping), to support the social and emotional wellbeing of Australian aboriginal children and young people

**DOI:** 10.1371/journal.pone.0312389

**Published:** 2024-12-30

**Authors:** Juli Coffin, Sharmila Vaz, Cheryl Kickett-Tucker, Helen Milroy, Craig Olsson, Meg Kirby, Lesley Nelson, Rob McPhee, Donna Cross

**Affiliations:** 1 Ngangk Yira Institute for Change, Murdoch University, Perth, Western Australia (WA), Australia; 2 The University of Western Australia, Perth, WA, Australia; 3 Edith Cowan University, Perth, WA, Australia; 4 University of Notre Dame, Broome, WA, Australia; 5 School of Education, Curtin University, Perth, WA, Australia; 6 The WA Centre for Health and Ageing (WACHA) and the School of Allied Health, The University of Western Australia, Perth, WA, Australia; 7 Michigan State University, East Lansing, Michigan, United States of America; 8 School of Medicine, Notre Dame University, Fremantle, Australia; 9 Centre for Child Health Research, The University of Western Australia, Perth, WA, Australia; 10 Perth Children’s Hospital, Nedlands, Perth, WA, Australia; 11 Faculty of Health and Medical Sciences, Division of Psychiatry, The University of Western Australia, Crawley, Perth, WA, Australia; 12 Faculty of Health, Centre for Social and Early Emotional Development, School of Psychology, Deakin University, Geelong, Victoria, Australia; 13 Centre for Adolescent Health, Melbourne Royal Children’s Hospital, Parkville, Victoria, Australia; 14 Department of Paediatrics, University of Melbourne, Parkville, Victoria, Australia; 15 The Equine Psychotherapy Institute/ Animal Assisted Psychotherapy International/ AWARE Therapy ^TM^ /Animal Assisted Psychotherapy /Animal Assisted Learning / Nature Assisted Psychotherapy / International Association of Human-Animal Interaction Organizations; 16 South-West Aboriginal Medical Service, Bunbury, WA, Australia; 17 Danila Dilba Health Service, Darwin, Australia; 18 Aboriginal Medical Services Alliance NT (AMSANT), Alice Springs, Australia; 19 School of Population and Global Health, The University of Western Australia, Perth, WA, Australia; 20 Telethon Kids Institute, Nedlands, WA, Australia; University of Oxford, UNITED KINGDOM OF GREAT BRITAIN AND NORTHERN IRELAND

## Abstract

Australian Aboriginal people experience stressors from inequalities across crucial social determinants, including deep and entrenched disadvantage and exclusion. The impact of unaddressed historical issues is pervasive and intergenerational. The disproportionate rates of Aboriginal youth suicide, juvenile detention and imprisonment highlight the inadequacy of existing social and emotional wellbeing programs and services for Aboriginal children and young people. There is increasing recognition in Australia that aligning social and emotional wellbeing interventions with Western values and conceptions of mental health is one of the main barriers to service uptake among Aboriginal people. This suggests fundamental questions remain unanswered about what type of services effectively address the complex constellation of social-emotional and wellbeing challenges arising from intergenerational poverty and trauma. Yawardani Jan-ga is an Aboriginal-led, operated, culturally secure, Equine-Assisted Learning (EAL) project designed by and with local Aboriginal young people, community Elders, members, and experts to address the complex constellation of social-emotional, spiritual and wellbeing needs of Aboriginal children and young people, aged 6–26 years, across multiple communities in the Kimberley region of Western Australia. EAL is a strengths-based learning approach where participants work with horses’ inherent characteristics to learn transferable life skills, such as communication skills, self-awareness, and emotional regulation, to promote social and emotional growth and wellbeing. Although EAL has been previously used with Aboriginal children and young people internationally, they are yet to be widely used with Aboriginal people in Australia. Here, we describe the three subcomponents of the Yawardani Jan-ga implementation science project and the planned Participatory Action Research and phenomenological approaches to capture the distinctive experiences of participants and the local communities where the intervention is implemented. We anticipate that findings will build an evidence base that informs policy and practice by understanding key intervention elements of social and emotional wellbeing support for Aboriginal youth, how to incorporate Aboriginal worldviews across different stages of interventions, and how to capture impact best using culturally secure methods.

## Introduction

Australian Aboriginal people experience stressors (e.g., poverty, frequent grief and loss, problematic substance use, trauma and violence resulting from genocide, child maltreatment) stemming from inequalities across crucial social determinants, including deep and entrenched disadvantage and exclusion [1–3]. The impact of unaddressed historical issues is pervasive and intergenerational. The 2005 Western Australian Aboriginal Child Health Survey (WAACHS) findings suggest that 35.3% of assessed Aboriginal children lived in households where at least one carer experienced forced separation from their natural family [4]. Carers from these households were nearly twice as likely to be arrested or charged with a crime and 1.61 and 2.1 times more likely to report alcohol and gambling issues, respectively. The Kimberley and Pilbara regions of Western Australia (WA) documented the highest rates of separation-affected households [4]. Not surprisingly, a quarter of Aboriginal children aged 4–17 were at increased risk of significant emotional or behavioural issues compared to their non-Aboriginal counterparts (~15%) [4].

Although the WAACHS data is almost twenty years old, the over-representation of Aboriginal young people in WA’s out-of-home care and youth justice systems suggests that past policies continue to impact Aboriginal Australian communities. Aboriginal young people account for an estimated 55% and 64% of all young people in out-of-home care and under youth justice supervision, respectively [5–7]. In 2020, the unequal positioning of Aboriginal children in Australia was shown by an incarceration rate 17 times higher than Australian youth of all other ethnicities combined [8]. Of concern is that although Aboriginal children comprise 6% of the Australian population aged 10–17, daily on average (between June and August 2020), they comprised 48% of those in youth detention [8]. An Australian study on a remote Indigenous community in the Fitzroy Valley, in WA, found the highest estimated rates of Foetal Alcohol Syndrome Disorder (FASD) reported in the literature to date (12%-19.4%) [9,10]. Young people with FASD are over-represented in youth justice settings; a prevalence of 36% was reported in a WA youth justice setting [11], the highest documented rate worldwide [12]. Further, medication use for conditions such as FASD and Attention Deficit Hyperactivity Disorder is alarmingly high in Aboriginal communities [13], as is the rate of suicide [14,15]. The rate of suicide in Aboriginal males in WA is twice that in non-Aboriginal males (48.6 deaths vs. 20.2 deaths per 100,000 population) and three times worse in Aboriginal females (21.0 deaths vs. 6.7 deaths per 100,000) [16]. Reducing suicidal behaviour and deaths by suicide in Aboriginal Australians is of significant concern in many communities and is a recognised bipartisan public health priority [16,17].

### The situation of aboriginal children and young people in the Kimberley region of Western Australia (WA)

The 2019 State Coroner’s Inquest into the deaths of a cluster of 13 Aboriginal children and young people in the Kimberley region of WA between November 2012 and March 2016 found that the cause of death for all 13 cases was a result of ligature compression of the neck (hanging), with 12 deaths as a result of suicide and in one case an open finding was made [18]. Five children were between 10 and 13 years old, three were aged 16 or 17, and the remaining were young adults aged 18 and 24 [18]. WA’s Coroner, Ms Fogliani, described the deaths as *“profoundly tragic*, *individually and collectively”*. The Inquest concluded that the underlying conditions, life events, and behaviours of these children and young people in the Kimberley signal the *prolonged exposure to intergenerational trauma and poverty*, including complex home environments, poor school attendance rates, little to no involvement with mental health services, and recurrent experiences of grief and loss, with several children belonging to families who had previously lost a close family member to suicide [18]. The term ’complex home environment’ is used to describe multifaceted factors that, when combined, diminish the ability to care for children and young people, hindering their ability to thrive and develop according to the broader expectations of Australian society. The Inquest laid bare the deep inequalities in remote Aboriginal communities in the Kimberley region. A recurrent theme throughout the Inquest concerned Aboriginal people’s desire to be consulted on matters that affect them and that purported solutions are not imposed upon them without consultation. Recommendations from the Inquest urged government agencies and services to acknowledge the importance of culture and inclusivity embedded in cultural healing for Aboriginal children, young people, and communities and to adopt a more collective and inclusive approach in developing and implementing programs targeting youth mental health in the Kimberley [18].

### Trauma and human development

Convergent developmental and neurobiological evidence suggests that prolonged or repeated exposure to adverse childhood events can lead to a traumatic response defined by often profound dysregulations of social and emotional functioning [1,2]. The capacity to learn and concentrate, develop trust and reciprocal relationships and use self-soothing or calming strategies to regulate behaviour is impaired in children who have experienced trauma, including intergenerational trauma [19,20]. Trauma leaves indelible footprints on the brain. Functional Magnetic Resonance Images show changes in the amygdala, hippocampus, corpus callosum, cerebellum, and prefrontal cortex [21,22].

The limbic system encompasses various brain structures situated above the brainstem and beneath the cortex, including the hippocampus and amygdala, which are integral to emotion and memory [23]. The amygdala, often called the brain’s alarm centre, is pivotal in the response and memory of fear and the regulation of emotions. When faced with a threatening situation, the amygdala initiates the body’s emergency survival mechanisms (fight, flight, freeze, or fawn response) [24]. Given its crucial role in our reactions to danger and the retention of emotional memories, individuals with a smaller-than-average amygdala may exhibit inadequate responses to stimuli. Chronic early-life stress is linked to alterations in the amygdala. When individuals with an atypically developed amygdala perceive a threat, they may experience intensified fear responses, anxiety, and flashbacks [25].

The hippocampus is responsible for forming new neural connections and storing memories, and it plays a role in regulating stress hormones. Traumatic events can cause this region to lose volume, leading to difficulties in memory recall. Damage to this area can impair the formation of new memories and increase stress hormone levels, further hindering memory recall [25].

The corpus callosum serves as a conduit, connecting the left and right hemispheres of the brain, and undergoes significant development in children aged three to six years. This development is related to attention and behavioural planning. In children aged six to thirteen, the corpus callosum continues to develop significantly in relation to language and memory [26], potentially explaining language impairments observed in some traumatised children [27].

The cerebellum, located at the back of the brain, is the most neuronally rich region of the brain and is involved in processes such as motor control, language, working memory, cognition, and emotion [26,28]. Decreased cerebellum volume has been observed in children with a history of trauma exposure [29]. Researchers suggest that reduced cerebellum volume may contribute to disturbances in language, working memory, and cognitive abilities, such as planning [26,28].

The prefrontal cortex is critical for mature cognition. Trauma significantly reduces activity in this brain region, leading to difficulties with memory and attention, emotional and behavioral regulation and inhibition, personality, decision-making, abstract reasoning, and learning [29,30]. Traumatised children and young people respond to their environment with limited access to the resources in their prefrontal cortex responsible for thinking, logic, analysis and problem-solving. Such alterations often manifest as problem behaviours in school, including aggression against self and others, difficulties establishing interpersonal relationships, substance use, depressive disorders, and suicide [19,20,23,31].

Experiences of trauma stay in the body [32] after the stressful or traumatic situation has passed. Trauma impacts the body’s physiological systems, particularly the hypothalamic-pituitary-adrenal (HPA) axis and the autonomic nervous system (ANS). The HPA axis regulates the body’s stress response, releasing cortisol during stressful situations. Chronic trauma exposure can dysregulate this system, leading to either hypercortisolemia (excess cortisol) or hypocortisolemia (insufficient cortisol), both of which can have detrimental effects on health [33].

The ANS, which controls involuntary bodily functions, also shows dysregulation in trauma survivors. The sympathetic branch of the ANS, responsible for the ’fight or flight’ response, can become overactive, resulting in chronic stress and anxiety. Meanwhile, the parasympathetic branch, which promotes ’rest and digest’ functions, may be underactive, contributing to difficulties in relaxation and recovery [34]. These physiological responses reflect how trauma can alter bodily functions, perpetuating a state of heightened arousal and stress.

The embodiment of trauma often manifests as somatic symptoms, including chronic pain, gastrointestinal issues, and cardiovascular problems. Studies have shown that trauma survivors are at a higher risk for conditions such as fibromyalgia, irritable bowel syndrome (IBS), and heart disease [32]. These somatic symptoms are not merely psychological in origin but are deeply rooted in the physiological disruptions caused by trauma. Not surprisingly, traditional talk therapy can feel unsafe to people who have experienced trauma as their systems are dysregulated and overwhelmed [32], mainly if the first traumatic experience occurred in childhood [35].

While essential life skills are vital to managing relationships at home, school, work, and outside interests, for Aboriginal children and young people from complex family environments, often a legacy of intergenerational trauma, violence, and distress become normalised reactions [36]. Childhood and adolescence provide a unique window of opportunity to create new pathways and rewire brain patterning [37]. Rapidly emerging modalities to enhance recovery and build resilience include mind/body, experiential approaches, and expressive therapies, like mindfulness-based self-compassion [38], trauma-focused Cognitive Behaviour Therapy [39], trauma-sensitive yoga [40], and expressive art therapies, including psychodrama [41]. These approaches de-emphasise verbal communication and help individuals establish therapeutic relationships, safety, and trust through non-verbal modes, which improve affect regulation and cognitive processing [42]. While there is a growing body of encouraging evidence on the effectiveness of these interventions for children who have experienced trauma, systematic reviews are yet to yield sufficiently strong evidence for any approach [43,44]. Further, these modalities have yet to be explicitly utilised with Australian Aboriginal children and young people and adapted to ensure cultural appropriateness and effectiveness.

Mental health systems and their policies and practices in Australia continue to reinforce colonial ideologies [45]. The persistence of colonial ideologies within Australian mental health systems is evident in systemic disparities, cultural insensitivity, inadequate Aboriginal staff representation within the mental health workforce, and marginalising policies and practices. Systemic disparities between Aboriginal and non-Aboriginal populations in mental health are rooted in the intergenerational trauma caused by colonisation, including the loss of land, culture, and identity, which has had a lasting impact on the mental health of Aboriginal peoples [36]. The Western biomedical model, which dominates mental health care in Australia, tends to overlook holistic approaches to wellbeing that are integral to Aboriginal cultures [46]. This model often pathologises Aboriginal people’s ways of knowing and healing, further marginalising these communities. Aboriginal representation within the mental health workforce limits the availability of such care and perpetuates a cycle of cultural insensitivity [47]. Mental health policies in Australia have historically marginalised Indigenous populations by prioritising Western paradigms of care. For instance, the National Mental Health Strategy, while acknowledging the need for culturally appropriate services, has been criticised for inadequate implementation and funding of Indigenous-specific programs [46]. The coercive practices within the mental health system, such as involuntary treatment and detention, disproportionately affect Indigenous Australians. These practices can be traced back to colonial control mechanisms that sought to dominate and regulate Indigenous bodies and minds [48]. The over-representation of Aboriginal people in child protection, youth detention centres and prisons across the country underscores the continued use of these coercive measures [5,7,8,49].

Western paradigms of care have been unsuccessful in making any significant inroads in addressing the lasting effect of intergenerational trauma on Australian Aboriginal children and young people and their families [6,50]. Engaging Australian Aboriginal children and young people with additional needs with mainstream health and welfare services is often complicated as those tasked with helping them (typically teachers, therapists, practitioners, and service providers) may be viewed with mistrust or suspicion [51]. Aboriginal children and young people with additional needs continue to face several barriers to accessing mainstream health services, including shame, stigma associated with help-seeking, limited knowledge of Western medical-based approaches, fear of the consequences of a diagnosis, fear that their children will be removed if they seek help for mental health issues, different cultural beliefs, and a shortage of culturally secure mental health services that are cognisant of ‘*Aboriginal ways of knowing*, *being and doing’* [17,48,50,52–55]. Cultural security goes beyond mere cultural awareness or sensitivity. It involves embedding cultural knowledge and respect into mental health services, ensuring that Aboriginal people feel culturally safe and understood when accessing mental health care [56]. Culturally secure mental health services are those that incorporate Aboriginal worldviews, values, and practices and that recognise the impact of historical and ongoing trauma on their mental health [56]. Although self-determination is central to providing mental health services, it is lacking in most Aboriginal policies and practices [17].

There is a growing understanding that support and services for Aboriginal Australians ought to align with the First Nations Framework of Health, honour the history of Aboriginal people, acknowledge their cultural practices and worldviews, be culturally secure, and work towards establishing trust between services, staff, and Aboriginal people, their families and communities [47,56,57]. Regrettably, there continues to be a dearth of culturally secure suicide prevention and intervention services and a workforce to meet the mental health needs of Aboriginal children and young people in WA’s Kimberley region. Recommendations from Coronial Inquests have been largely ignored, as have local community calls for actionable change. There is an urgent need for new, innovative, culturally secure ways to engage Aboriginal children and young people with additional needs in the Kimberley region.

### Equine-assisted learning: A culturally responsive approach to address the social-emotional wellbeing (SEWB) needs of aboriginal children and young people with additional needs

Equine-Assisted Learning (EAL) is an experiential learning approach that incorporates safe relational experiences with horses to foster clients’ life skills, social and emotional development, growth, and learning [58,59]. Skill development and learning in EAL arise through the repeated practice of relational skills gained through planned learning activities (equine experiences) involving equine-client interactions and post-activity reflections guided by certified practitioners [59,60]. Essentially, the horse is an *‘active partner’* to support the client in developing or bolstering life and/or professional skills (e.g., leadership and team building). One of the ways horses contribute to client change is by facilitating a biofeedback loop (providing a behavioural response or *‘unique Feedback’*) entailing sensory integration, relational skills, self-regulation, social-emotional wellbeing skills (SEWB), positive coping and adaptation to diverse stressors [51,58,61–63]. EAL thus aligns with traditional understanding and empirical findings that acknowledge that connecting with creation in all its forms is fundamental to the process of healing in the Aboriginal culture [64–66] and is *consistent with Aboriginal ways of knowing*, *being and doing* [66,67].

Aboriginal people have an inclusive outlook on how the systems fit together and interact [68–70]. They do not believe in power relations over other forms of living or non-living existence [71]. Such a worldview understands the individual, the collective, and the universe as an interconnected web of being [68], which is congruent with field theory embedded in the EAL approach that *“everything is interconnected”*[58] [p. 38]. Aboriginal understanding places the *‘Spirit’* at the centre of knowing and recognises its innate existence in all life forms. The equine (horse), like any other of Creator God’s making, has a *life force or a Spirit* and hence is sacred. Thus, including a horse as a *‘partner’* in the experiential learning team aligns with Aboriginal ways of knowing and being [72].

*‘Ways of doing’* convey Aboriginal people’s ways of knowing and being [67]. EAL’s experiential, hands-on nature encourages learning through concrete outdoor activities *(doing)* and is less reliant on spoken language, thus aligning with Aboriginal ways of learning and expressing themselves [58]. Therefore, an EAL program may appeal to tactile learners and those for whom verbal modes of communication are not the primary mode or for whom, developmentally, such capacities are still emerging [58,66,73–75]. EAL is aligned with the ecopsychology approach to healing the more fundamental alienation between the recently created urban psyche and the age-old natural environment [76]. Such an ecopsychology approach perfectly aligns with Aboriginal people’s innate connection to the land [58,73,77,78].

Horses are social, perceptive, sensitive, and intelligent animals [58]. As herd animals, horses need contact with others for survival and belonging [58,79]. Further, horses have an instinctual communication system primarily based on body language [59,80]. Diverse EAL models propose that one of the ways that horses can contribute to client change is when the horse mirrors or gives unique feedback to the unique client’s approach, nervous system, emotion, and behaviour (affective mirroring), which serves as a powerful tool for learning [58,79,81]. When culturally adapted to suit Aboriginal people’s ways of knowing, being and doing, EAL sessions can afford Aboriginal young people a safe place to experience something new, to learn and reflect, enhancing the client’s potential experience of belonging, and increase their capacity for mentalising (i.e., attention to mental states, including the mental states of others, particularly in explanations of behaviour). Overall reflective functioning is increased (i.e., awareness of the nature of mental states in oneself and others, the mutual influences at work between mental states, and behaviour through an exchange with the horse) [58,66]. Engaging in activities with a horse in a natural outdoor space (on country) rather than a conventional therapy room may reduce the perceived dominance of the practitioner and perhaps offer Aboriginal young people a sense of neutrality [58,66,73] to explore their feelings and emotions in a safe and non-judgemental space [58,82–84], learn roles and responsibilities of caring for one another–which is consistent with Aboriginal peoples’ of being and doing [66,67,69].

Since each horse is unique, culturally adapted EAL sessions (included in Yawardani Jan-ga EAL) can be structured to offer young people the autonomy to choose a horse like themselves or someone in their life, thereby encouraging self-determination [58,59,66]. Young people can select the horse they partner with on the day of the EAL session and the flow of activities [66]. For example, a low-twitch horse may be drawn to a young person who feels tired and listless. Further, individual sessions in Yawardani Jan-ga are tailored to meet each young person’s unique needs [59], with consecutive EAL session schedules following progressive build-on skills [66]. Thus, Yawardani Jan-ga EAL offers a creative, flexible, and innovative modality to personalise sessions based on individual needs and continuing progress [59,67,85]. These adaptive elements make EAL an essential means to contribute to the vision of equitable health service provision for Aboriginal young people. Further, given that most of the trauma experienced by Aboriginal young people is through personal relationships (immediate, social, and system-based), the EAL approach with an animal offers a safe alternative that can assist in restoring the basis for relationships that were altered with trauma; giving the young person a safe space to learn to trust people again. Finally, as a relatively new alternative approach, EAL has no established prejudice and may appeal to Aboriginal children and young people across WA’s Kimberley region who are reluctant to engage with traditional Western therapy [66,73,86].

### Evidence for EAL’s efficacy in Aboriginal children and young people with additional needs

Findings from existing scoping and systematic reviews across the broader Equine-Assisted Services space suggest that Equine-Assisted Learning approaches (EAL) can successfully engage Aboriginal children and young people with additional needs who have not previously responded to traditional therapeutic interventions or those who experienced sexual abuse or were exposed to problematic parental substance use [74,87–90]. Reported outcomes include increased prosocial behaviours [91,92], confidence and self-esteem [51,93,94], self-awareness [66], self-control and emotional regulation [82,93,95] and reduced anxiety [92,94]. Participants with a history of trauma have commented on the safety and comfort horses provide [79]. Others reported improved acceptance, trust, respect, warmth, collaboration, belongingness, connectedness, and mastery [80,96].

Only one scoping review addressing EAL has explicitly included studies with Aboriginal children and young people aged 6–26 in the sample [74]. The review included three Equine Assisted Learning (EAL) programs and one adapted Equine Assisted Psychotherapy (EAP) program that explicitly targeted Aboriginal participants with additional needs, with two programs being from Canada [60,72] and one each from the United States of America, US [97] and Australia [66]. All four programs partnered with local Aboriginal communities during intervention development and implementation and were trauma-informed and spiritually grounded. All reported positive findings for cultural safety, belonging and connectedness to culture and spirituality [74]. Reductions in anti-social behaviours [66], improvements in SEWB [60], and social adjustment at 12 months post-intervention were also evident [97]. Further, the Australian EAL program (called *’Nguudu Barndimanmanha’* or Horses Making Good) included modules on teaching leadership and problem-solving skills and reported improvements in participants’ communication skills and ability to cope with emotions and stress for Aboriginal young people 6–25 years [66]. Thus, EAL programs show promise in achieving therapeutic and learning goals in young people aged 6–26 with additional needs, including Aboriginal people with a history of trauma/abuse, mental health and learning disability [74].

### Gaps in the EAL evidence base

Although individual EAL programs show promise in complex population groups with additional needs, findings from systematic, scoping reviews and meta-analyses that typically combine studies irrespective of the scope of practice (namely, combine EAL, EAP and Hippotherapy studies) have consistently critiqued the evidence base for lacking theoretical and methodological rigour and interchangeable use of terminology to describe programs [74,90,98]. The summative presentation of outcome effects in systematic reviews and meta-analysis makes parcelling out the contribution of individual intervention modalities and discernment of active intervention components challenging. Further, small sample sizes, absence of control or comparison groups or randomisation, limited detail about intervention programs and their theoretical underpinnings, and paucity of psychometrically robust measures are commonly reported flaws [74,90,98,99]. The fact that until 2023, only four studies have included Aboriginal young people in their sample suggests more work in this population is needed [74]. There is a need for standardised and culturally secure intervention manuals to safeguard implementation fidelity and psychometrically robust and culturally secure measurements of meaningful changes in outcomes over time [66,100,101]. Future studies are needed to establish the appropriateness of EAL in Aboriginal youth and the ecological validity of outcomes beyond the EAL program, especially in light of the high dropout rate amongst participants from unstable households [74]. In addition, understanding the dose required to establish program effectiveness is an essential consideration for financial feasibility.

The Yawardani Jan-ga project attempts to bridge the gaps in the scientific evidence base, respond to recommendations of the Coronial Inquest and address community needs in the Kimberley by co-designing, co-implementing, and co-evaluating with the local community members a strengths-based, evidence-informed, culturally secure, adaptive, EAL program to build local Aboriginal young people’s SEWB skills and help youth establish a solid connection to self, community, and country.

## Overview of the Yawardani Jan-ga project and its objectives

**Fig 1:**
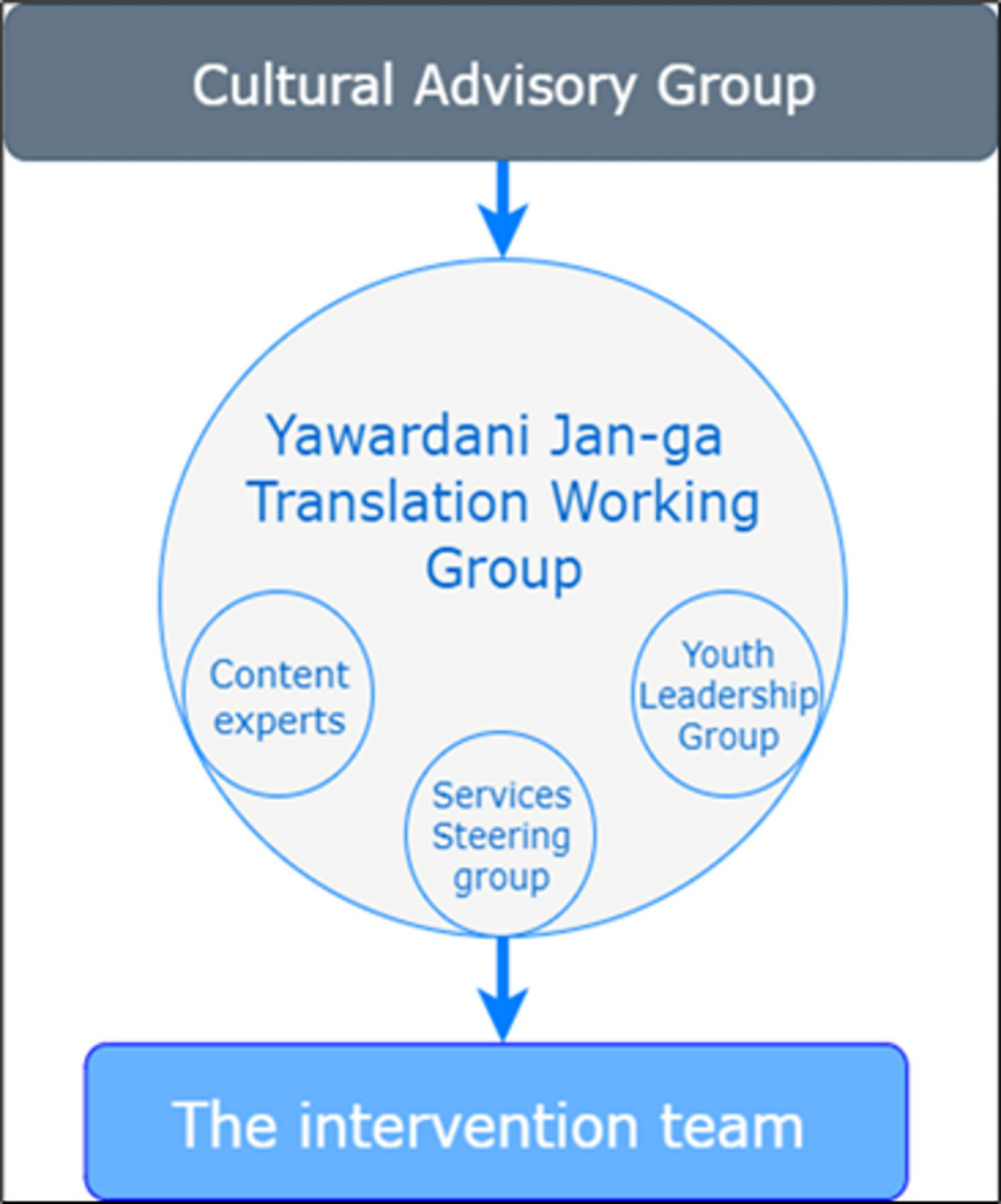
Yawardani Jan-ga translation working group.

**Fig 2:**
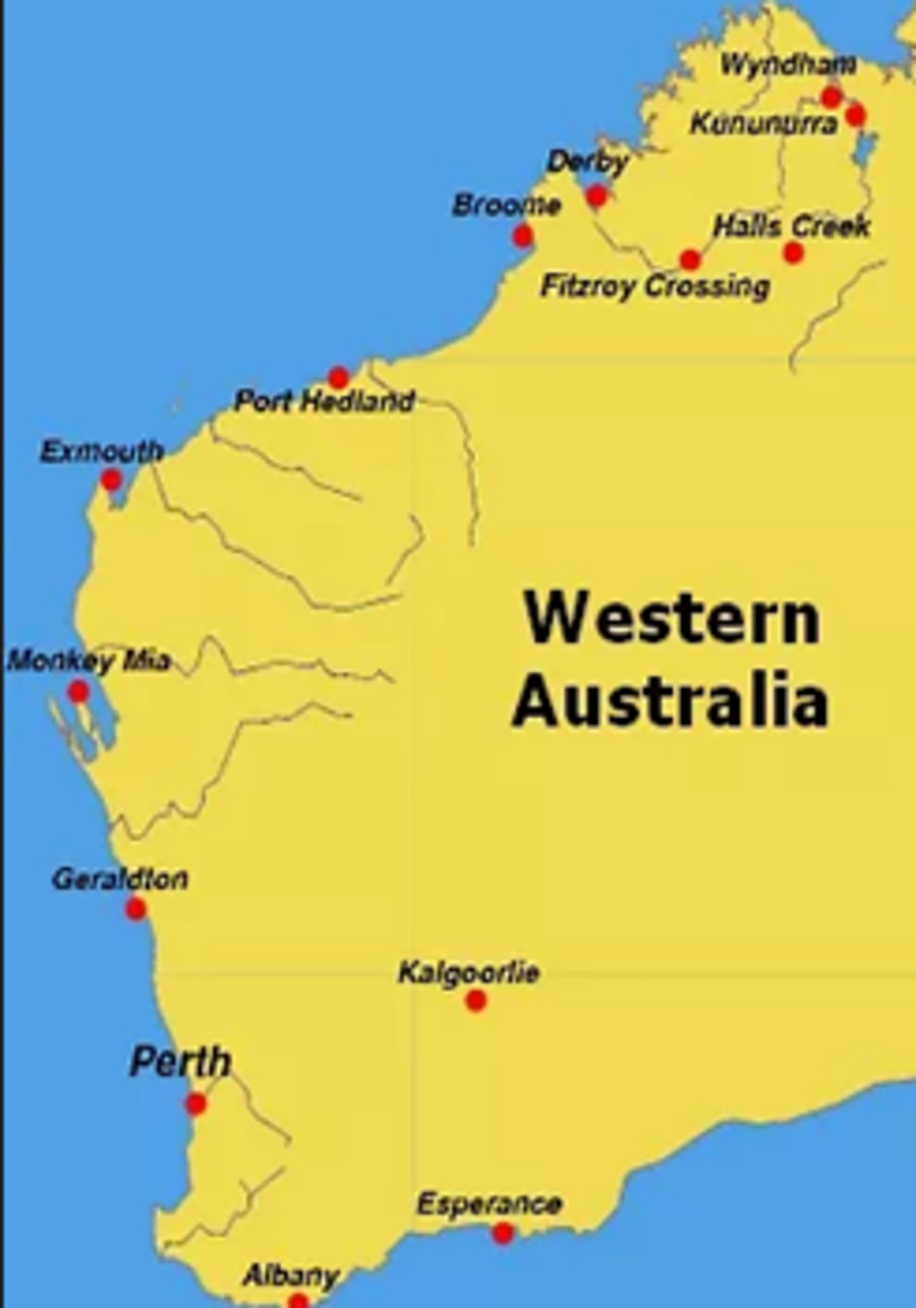
Map of Western Australia. Reprinted from https://www.kimberleyaustralia.com/map-western-australia.html under a CC BY license, with permission from Kimberly Australia, original copyright 2006–2024 Birgit Bradtke.

The Yawardani Jan-ga project draws on Aboriginal people’s strong historical ties to horses and the Kimberley region’s rodeo culture and connection with pastoral industries. It is an Aboriginal-led and operated, culturally secure, Equine-Assisted Learning (EAL) service designed by and with local Aboriginal young people, community Elders, members, and experts. It is designed to address the complex constellation of cognitive, emotional, behavioural, and spiritual issues experienced by Aboriginal children and young people across multiple communities in the Kimberley. The Yawardani Jan-ga project has three subcomponents, the objectives of which include(Figs 1 and 2), (S1 and S2 Figs).

- ***Objective 1*: *Grow and build the capacity and confidence of the local Aboriginal Equine-Assisted Learning (EAL) workforce and build research capacity across Kimberley communities to address the SEWB needs of Aboriginal children and young people*.**
- ***Objective 2*: *Effectively implement the Yawardani Jan-ga EAL intervention across multiple Kimberley sites*.**
- ***Objective 3*: *Evaluate the implementation and impact of the Yawardani Jan-ga EAL project on the SEWB and life skills and plan for its sustainability if found effective*.**

## Methods

### Design

Yawardani Jan-ga is an implementation-science project. The project design emerged after extensive consultation and engagement with community members and local services in the Kimberley region of Western Australis (WA) [92] and includes Participatory Action Research (PAR) [102] and phenomenological approaches [103] to capture the distinctive experiences of participants enrolled in the EAL intervention and the local communities where the intervention is implemented [104]. These methodologies offer flexible yet structured approaches to build the faith and trust necessary for collaboration and partnership and integrate local communities’ and services’ experiences and expertise [56,104,105]. Yawardani Jan-ga project adopts a co-design approach from design planning to implementation and evaluation. This approach ensures that the experiences, values, and worldviews of Aboriginal people (including children and young people) are at the core while facilitating the pooling and coordination of community resources [95].

### Ethics and identified risks

Continued successful engagement with community members, Aboriginal leaders and local services will be critical for the success of the Yawardani Jan-ga project. Aboriginal leadership will ensure this project is culturally secure from conception to dissemination. This includes methods for measuring impact among participants. For example, data collection processes have been selected to minimise distress by acknowledging potential participants’ *’high-risk’* profiles. Similarly, the planning and outcomes of the EAL Intervention are based on neurodevelopmental concepts, and our approach is trauma and culturally informed.

Two documents produced by the National Health and Medical Research Council of Australia underpin the design of this research: Ethical Conduct in Research with Aboriginal and Torres Strait Islander Peoples and Communities: Guidelines for Researchers and Stakeholders [106] and Keeping Research on Track II: A companion document to Ethical conduct in research with Aboriginal and Torres Strait Peoples and Communities: Guidelines for researchers and stakeholders [107].

Ethical approval for this research has been granted by the WA Aboriginal Health Ethics Committee [Reference: 926] and the Kimberley Aboriginal Health Planning Forum, Research and Evaluation Subcommittee [Reference: 2019–007], The Department of Education, Government of Western Australia [Reference: D20/0184973], The Catholic Education Western Australia [Reference: RP2019/36] and The Western Australia Police Force [Reference: T539]. Refer ST3. Animal ethics for using horses in this research has been sought from Murdoch University’s Animal Ethics Committee [Permit No. T3404/22]. The protocol is prospectively registered with the Australian New Zealand Clinical Trials Registry [ACTRN12619001675112; Universal Trial Number: U1111-1241-7349]. We acknowledge the COVID-19 context in which this project is being implemented.

### Community involvement and methodological engagement

Acknowledging the power and knowledge in Aboriginal communities, the overarching approach to Yawardani Jan-ga is Aboriginal leadership and methodological involvement through its investigators, governance, EAL-intervention development, implementation and evaluation structures [108]. A Cultural Advisory Group governs Yawardani Jan-ga with representation from Elders across five cultural blocs [109].

*The Cultural Advisory Group* ensures that Aboriginal experiences, values, and worldviews remain at Yawardani Jan-ga’s core. Additionally, working groups of *Content Experts*, *Local Services*, *and Youth Leadership* will be established, with nominated members from each group to inform a *Translation Working Group*. Guided by the Cultural Advisory Group, the *Translation Working Group* will assist in developing culturally responsive evaluation processes, frameworks, and strategies for translating findings into embedded practice and sustainable community partnerships.

*The Translation Working Group* comprises 10–12 members from Kimberley, Broome Regional and Derby Aboriginal Medical Services, headspace, Kimberley Police District Office, Catholic Education Broome Office, and the Western Australian (WA) Education Department Broome Office, as well as community members (youth and Elders).

The Translation Working Group allows community members, youth, local services, and content experts to respectfully work together, share and learn while building solid connections. This multi-level community involvement in Yawardani Jan-ga will ensure that Aboriginal people not only support the SEWB of young people in their communities but also make a significant contribution to the research process, including guiding how to implement the EAL program, informing how SEWB is expressed and manifests in Aboriginal children and young people, and how to monitor progress among Yawardani Jan-ga participants over time (follow-up). Ongoing stakeholder and community consultation processes are well established in Broome and Halls Creek and safeguard community readiness in both sites and high-quality input into the delivery planning. In Broome, for example, a local referral network akin to a *’no wrong door’* approach has been set up to intervene early and prevent the decline of wellbeing of Aboriginal youth (S3 Fig). This means Aboriginal children and young people who need diverse levels of SEWB support for various reasons, including bullying, trauma, exposure to domestic violence or substance use, can be referred to Yawardani Jan-ga. Sources of referral can include WA Police and Juvenile Justice; Youth services such as the Police and Community Youth Centres and Alive and Kicking Goals; Aboriginal community-controlled health services: Broome Regional Aboriginal Medical Service, Derby Aboriginal Health Service and Kimberley Aboriginal Medical Service; Mental health services such as Headspace and Kimberley Mental Health and Drug Services Child and Adolescent Health Service (Broome and Derby); and the Department of Education and Catholic Education primary and high schools.

### Yawardani Jan-ga EAL intervention development

The Yawardani Jan-ga EAL intervention builds on an earlier EAL program called *’Nguudu Barndimanmanha*’ (Horses making good) that targeted the SEWB of Aboriginal young people with additional needs, living in the MidWest region of WA, namely, Geraldton city (Fig 2) [66]. The *’Nguudu Barndimanmanha’* EAL program incorporated EPI Horse Wisdom Program elements to thematically organise sessions [59,66]. The *’Nguudu Barndimanmanha’* EAL team partnered with multiple service providers in the Geraldton community, including the Department of Child Protection, local primary and high schools, and the Regional Aboriginal Medical Service and was accessed by 270 Aboriginal young people aged 6–26 years, in blocks of 6–10 weeks over one calendar year. Parents and teachers reported improvements in self-regulation, self-awareness and socialisation skills. Feedback from young people demonstrated the feasibility, acceptability, cultural appropriateness and preference of EAL over room-based interventions previously experienced by the participants [66]. The Yawardani Jan-ga EAL program scales up its predecessor to more systematically and rigorously evaluate the impact of EAL on the SEWB needs of Aboriginal children and young people aged 6–26 years across several Kimberley sites. It also measures whether EAL intervention effects are sustained after young people complete their regular EAL sessions.

### Theoretical underpinnings of the Yawardani Jan-ga EAL program

Yawardani Jan-ga EAL is a trauma-informed, recovery-oriented, culturally secure program underpinned by Aboriginal knowledge, concepts, and bioecological (collective) view of health theories of child development [56,110–113]. Similar to its predecessor, the Yawardani Jan-ga EAL sessions thematically draw from the Equine Psychotherapy Institute’s (EPI) Horse Wisdom Program [59], the neuroscience of attachment [84], the Neuro-sequential Model of Therapeutics (NMT) [19,20] and the Six Rs of Positive Development [114].

The Neuro-sequential Model of Therapeutics (NMT) outlines how trauma affects brain regions sequentially, beginning with the lower, more primitive parts of the brain (e.g., brainstem) and moving to higher, more complex areas (e.g., cortex). In line with the NMT, session order has been carefully planned to enable sequential resourcing in a manner that replicates neural organisation and development (ST1) [19,20]. Activity content is built around repeated somatosensory regulatory experiences (e.g. repeated brushing of horses) in a relationally enriched environment (e.g., building trusting and healthy connections with horses and EAL-practitioners) that allow emotions to become less threatening and overwhelming, leading to emotional and behavioural change. As participants achieve greater regulation and relational security, activities that stimulate higher brain functions are introduced. These include problem-solving tasks with the horses, such as navigating obstacle courses, which engage the cortex and enhance executive functioning. Emphasis is placed on post-activity reflections facilitated by highly skilled Aboriginal EAL practitioners, with dialogue focusing on reflecting on the horse’s nonverbal communication or biofeedback.

The Six Rs of Positive Development—relationship, repetition, regulation, respect, reward, and routines—are foundational principles for fostering adaptation and recovery in trauma-informed care [115]. These principles are operationalised through specific horse-assisted activities in the Horse Wisdom Program context. For instance, building a trusting relationship with the horse and the EAL practitioner can provide a safe and secure environment for participants (relationship). Repetitive, rhythmic activities, such as grooming or leading the horse, can help regulate the nervous system and promote a sense of calm (repetition and regulation). Ensuring that interactions with the horse are respectful and acknowledging each participant’s efforts can foster mutual respect and self-esteem (respect and reward). Finally, establishing consistent routines in the sessions can create a predictable and safe therapeutic environment (routines).

### The equine psychotherapy institute (EPI) partnership with the Yawardani Jan-ga EAL program

Yawardani Jan-ga EAL program places a cultural lens on the EPI Horse-Wisdom Program [59] and other elements, such as the EPI Theory of Change, where horses are included in the experiential learning process as potential change agents. Similarities can be found with the four pillars of EPI practice methodology, including:

- Phenomenology and the Phenomenological Method of Inquiry [116];
- Humanistic, Experiential, and Relationally oriented Psychotherapy [58,117–119];
- Dialogic or I-Thou relating [120]; and
- Awareness and Mindfulness [121].

### Yawardani Jan-ga EAL session plan and delivery

#### Session plan

As a health promotion, prevention, and early intervention treatment service, Yawardani Jan-ga EAL sessions are designed to have the greatest impact on Aboriginal children and young people’s SEWB [122]. Yawardani Jan-ga EAL sessions address the needs of *both ‘*Aboriginal youth with additional needs and those with leadership and capacity needs’. EAL sessions for Aboriginal young people are of two types: (i) *SEWB focussed sessions* (achieved a minimum attendance of 10 sessions as per protocol (10 SA), with each session 60-90-minute long and delivered weekly). Yawardani Jan-ga has also established three tiers (Education Department classification) of re-referrals, as evidence indicates 10 sessions may not be enough for young people with severe or complex needs); and (ii) *Leadership and Capacity focussed sessions* (typically a minimum of 5 to 8 sessions, with one 60-90-minute weekly session). Please refer to the primary participant section of the manuscript for definitions of each category of participants.

Yawardani Jan-ga EAL sessions are adaptive [85], person-centred [123], strengths-based and solution-focused [124]. As an*’adaptive’* program, the number of sessions and the time over which sessions are delivered can be adjusted to meet each young person’s unique challenges and needs to optimise developmental outcomes [85]. There is a temporal progression of activities over sessions, meaning that skills and abilities gained in one EAL session will be built on to gain new skills and abilities in subsequent sessions.

Furthermore, activities focus exclusively on each young person’s *‘Here & Now experience’* without drawing attention to their early life experiences (e.g., How did the horse react to your movements? What is the horse trying to tell you? How is the horse telling you this?). EAL practitioners use the phenomenological method of inquiry, mentalising, and reflective functioning techniques to help participants understand the horse-client interaction [63,82,125]. These techniques are hypothesised to help the young person become more *aware* of physical sensations generated by their bodies and how their body reflects their feelings, thoughts, beliefs, needs and wants and create a safe space to experience and express emotions and thoughts. As sessions progress, the practitioner assists the young person in reflecting on old ’unhealthy’ unhelpful behaviours and creates a dialogue around how the participant might respond differently in the future [63,126]. Each participant moves through sessions at their own pace, so the total duration of the program is adjusted to meet each young person’s needs and challenges and may be more or less than the recommended 10 sessions. Details on the open-door referral process and data collection schedule are presented in S3 Fig.

#### Session delivery

Trained Aboriginal Yawardani Jan-ga EAL Practitioners deliver sessions in an off-campus EAL site. Sessions typically last 60–90 minutes during school hours (9 am–3 pm) and occur once weekly over a 10-week school term. EAL sessions are planned to enrol a new cohort of EAL participants for each of the four school terms (i.e., every 10 weeks), with some cohorts including participants from earlier cohorts. Participants are picked up from and dropped off at school as part of the EAL service, and a snack is provided after the session. Depending on the participant’s needs, sessions can be delivered one-on-one (one horse, one EAL practitioner, one young person), in dyads, in a small group (> 3 and < 6 participants), or in a large group (6 or more participants), or with more than one horse. Sessions are structured to include:

- Meet and greet, check-in, and feedback around participant’s current emotional state (approximately 10 minutes).
- Participant and EAL practitioner select a suitable horse for the session.
- Participant enters the set area with the horse and the EAL practitioner to undertake an activity (approximately 40–50 minutes).
- Closure and goodbye to the horse/horses.

### Addressing the Yawardani Jan-ga Project’s Objectives

- ***Objective 1*: *Grow and build the capacity and confidence of the local Aboriginal Equine-Assisted Learning (EAL) workforce and build research capacity across Kimberley communities to address the SEWB needs of Aboriginal children and young people*.**

To address Objective 1, the Yawarandi Jan-ga project embodies two significant capacity-building opportunities for the communities where the EAL program is implemented. First is the opportunity to develop and provide employment for a workforce of front-line EAL practitioners able to deliver culturally safe early intervention for emotional and behavioural concerns. The second is the opportunity to develop and provide employment and career opportunities for Aboriginal researchers beyond this project.

#### Preparatory phase (6 months)

This involves community consultations for project sites as nominated by the Cultural Governance Committee. These consultations ensure community need, readiness for implementation, and alignment of the implementation processes with the local context, including referral pathways, coordination mechanisms, and identification of candidates for EAL traineeships.

#### Capacity building and training

Thirty EAL practitioners across the Kimberley region will be trained throughout this project (across Broome, Halls Creek, and Derby to conduct outreach services). Potential EAL participants will be identified through community consultations with Elder groups and other organisations in contact with Aboriginal young people. These Aboriginal EAL practitioners will receive the EPI-endorsed–Yawardandi Jan-ga Aboriginal-specific EAL training package and will be recognised by the Equine Psychotherapy Institute through the accreditation process led by authors Kirby and Coffin. These practitioners will receive continuous supervision and mentoring from the first author (Coffin) to ensure they have access to support, expertise, skills, and perspectives related to their development in the EAL space. As a result, they are well prepared to deliver the EAL program in their respective communities.

*Expansion to other sites across the Kimberley region*. Over time, in addition to training local community members in EAL, the project will also offer opportunities for advanced training in EAL, thereby creating a pool of local Kimberley-based accredited trainers and enabling mentoring and post-training support for novice EAL practitioners beyond the lead author. These skills could also create an opportunity to expand EAL services to family groups, acknowledging that individual support may be insufficient to promote the wellbeing of youth in specific situations [110–112]. EAL traineeships will be free to local community members at no cost at each project site.

#### The process of recruiting EAL practitioners

Recruitment will follow comprehensive consultation with community Elder groups to identify respected and trusted local members and give communities ownership of their issues and solutions. The lead author, an Aboriginal-certified EAL practitioner, will co-deliver the accredited training of new EAL trainees. The training will occur in the Broome project site in three one-week blocks across three months (15 days total, 120 hours) in the first half of Year 1 of this project.

The need for Aboriginal-specific EAL Training was identified by the first cohort of Aboriginal community members in March 2019, who, when training in the mainstream Equine Psychotherapy Institute (EPI) Model, expressed challenges in understanding the European-based theory and language. The lead author (Coffin) and EPI founder and author (Kirby) together agreed on the need for culturally secure training, where the training of Aboriginal EAL students is delivered by Aboriginal Trainers, with a curriculum including Aboriginal culture, language, and practices. This was the beginning of the Yawardani-Jan-ga training program. In collaboration with the EPI, the Yawardani Jan-ga team developed an Aboriginal-specific EAL training package that draws from Aboriginal knowledge, concepts, and theories of child development and trauma-informed practice [56, 110–113]. The Yawardani Jan-ga EAL training package consists of a Teaching Curriculum, Trainer and Trainee manuals, and video-based training content aligned with the Aboriginal ways of learning, being, and doing. Developing an Aboriginal-specific Yawardani Jan-ga EAL training package assisted in implementing standard operating practices and ensured Aboriginal practitioners deliver safe, ethical, and effective EAL services across the Kimberley region.

After training completion and certification, the trained EAL practitioners will be offered employment opportunities through Yawardani Jan-ga (six part-time positions available) or through integrating the position within local services. In addition, to ensure consistency in consent processes and data collection, storage, and processing, all Yawardani Jan-ga employees will undergo specific and standardised training in ethical conduct and research methods.

For ongoing support, the EPI will be further engaged to develop future EAL/EAP training should the need arise and will provide supervision to the lead author and more advanced Aboriginal EAL practitioners, continuing the support and the development of the future localised Aboriginal EAL capacity and capability.

#### Build research capacity across Kimberley communities to address the SEWB needs of Aboriginal children and young people

Yawardani Jan-ga will also offer community members continuous learning opportunities throughout the project, including the opportunity to develop applied research and evaluation skills. It will also build the capacity of Aboriginal researchers through formal university channels. By developing research skills in local Aboriginal people, the program will tap into professional skill sets, family connections, and community dynamics, which are critical for brokering relationships between researchers and community members and enable effective communication of research processes and findings.

During the implementation phase (Objective 2), Yawardani Jan-ga trained EAL practitioners will receive ongoing skill development by facilitating sessions under the supervision of the lead author and by reviewing notes, photos, and video footage as recorded during sessions. The project aims to ensure sustainable availability and accessibility of EAL programs across the Kimberley region through strategic partnerships, embedding EAL in Aboriginal medical services, leveraging the National Disability Insurance Scheme funding (NDIS) and exploring social enterprise models [127].

- ***Objective 2*: *Effectively implement the EAL intervention across multiple Kimberley sites*.**

#### Enrolment phase (30 months)

The key intervention-delivery considerations identified in Objective 1 will underpin the implementation of Yawardani Jan-ga EAL across sites. Implementation will be staggered, with new sites added at different time points in the first 12 months of the project. Implementation commenced in Broome, followed by Halls Creek and an additional site (to be determined in consultation with the Cultural Governance Group). An outreach service delivery modality will also be implemented to enable remote communities in the Kimberley region to access EAL. It is expected that diverse models of service integration with project partners (e.g., Aboriginal medical services, schools, police) will be tested and trialled during the project, resulting in full integration through the development of agreements that enable efficient use of community resources in each site.

#### Design

To ensure a culturally sensitive approach to implementation evaluation, we propose to take an entirely phenomenological approach to understand the program’s impact on life skills and social and emotional wellbeing (SEWB) of children and young people. The decision to focus on qualitative methods, including interviews with participants and behaviourally coded video recordings of human-horse interactions, was taken to address two primary limitations of the field. The first is the inadequacy of available psychometric measures to capture the complexity and nuances of therapeutic interactions within the EAL program. The second is the inadequacy of available psychometrics measures to capture the world views and experiences of Aboriginal children and young people in culturally sensitive ways.

For these reasons, we propose to run a multiple-site, longitudinal qualitative program implementation evaluation design, with pre-intervention (Referral documentation), during the intervention (after sessions 5 and 10) and post-intervention (3-month, 6-month and 12-month) data collection points. The intervention includes access to the Yawardani Jan-ga EAL program. The EAL program will be delivered over 30 months at each site (as detailed in the enrollment phase above).

#### Participant groups

Community and stakeholder consensus determined the criteria for the inclusion of participants.

***Primary Participants*: *Inclusion criteria***: (i) Aboriginal young people aged 6–26, who are considered ‘*to have additional needs’* or to be *‘emerging leaders’*, and (ii) living in Broome, Halls Creek or Fitzroy Crossing at the time of consent; and (iii) willing to commit to a minimum of 10 sessions if referred for the *SEWB focussed EAL program* or a minimum of five sessions if referred for *‘Leadership and Capacity’ focussed EAL program*. The inclusion of young leaders recognises that even young people who seem to be ’doing well’ can take their own lives by suicide and experience enormous pressure as they navigate their two worlds. The term ‘*additional needs group’* refers to young people whose academic, social and/or emotional attributes are a barrier to student engagement, educational achievement, and social integration within the school community. This could include students with one or more risk factors, including irregular school attendance, low socio-economic status, family structures, medical/biological/genetic/cognitive factors, and environmental factors (school, family, and community).

***Exclusion criteria***: (i) non-Aboriginal children and young people; (ii) Aboriginal young people older than 26 years; and (iii) Aboriginal children and young people 6–26 years with a severe physical disability. The program will not initially have facilities to accommodate those with more complex disabilities, such as mobility, in the current round. The program will endeavour to find funding to facilitate this in the future.

***Secondary Participants*:** Secondary participants include parents/carers of participants, staff from agencies referring participants (e.g., teachers and/or principals, key informants from the community, police officers, mental health workers, and health providers), Aboriginal community leadership, and the EAL practitioners working with participants.

### Sampling

***Primary Participants*** Given the high truancy rates, cumulative risk factors and mobility of the families across the implementation sites in the Kimberley, it is estimated that over the five years of the project, approximately 600–700 young people will be invited to engage with the project. This represents approximately 10%-13% of the total Aboriginal population aged 6–26 years in the Kimberley. This estimate considers the staggered implementation of Yawardani Jan-ga across sites and the five weeks, ten weeks, 3-month, 6-month and 12-month follow-up data collection points for each participant.

***Secondary Participants*:** Purposive sampling will be used to recruit parents/caregivers of primary participants, referrer staff from local services and EAL practitioners employed by Yawardani Jan-ga to deliver the program across sites.

### Recruitment

***Primary and Secondary Participants*:** Recruitment of primary participants will occur every ten weeks and coincide with the commencement of the school term. The first recruitment commenced in February 2020 and will continue until December 1, 2024. Participants will be generally enrolled in one ’block’ of EAL intervention, with each ’block’ corresponding to a school term in the academic timetable. Whilst enrolled in the intervention, participants attend weekly EAL sessions to achieve the desired intervention outcomes. However, a key feature of EAL is its flexibility in being personalised or adapted to meet the specific needs of each young person.

Potential participants can be referred to Yawardani Jan-ga by staff from local organisations in Broome and Halls Creek (e.g., schools, youth service, medical services, Aboriginal community-controlled health services, judicial system, police) using a study-specific referral form. Recruitment packs (containing poster, flyer, referral form, participant information and consent forms) will be distributed through the referral network.

#### Consent process

As the program works with young people 6–26 years of age, there are varying levels of consent for participation in Yawardani Jan-ga:

***1*. *Primary participants aged 6–15 years old referred to the program***: In the case of this subgroup of children, written consent of parents/carers/ refer will be obtained. Once their parent or guardian consented to their participation, the child will be asked for verbal assent when they arrive at the first session. When Yawardani Jan-ga receives a referral from a local organisation, the Investigator or a Yawardani Jan-ga Aboriginal team member will arrange to visit the parent/carer or current legal guardian to discuss the project, answer questions, and ask for consent for the young person to be involved. During the consent process, the Principal Investigator or a Yawardani Jan-ga Aboriginal team member will clearly explain the research to the young people and the parent/carer by going through the Participant Information Sheet. This includes explaining EAL, what to expect in a session, the potential benefits and risks, what kinds of data will be collected, when (and if) that data will shared and with whom. The investigator or team member will check the parent/guardian and young person understand the information provided, have the opportunity to ask any questions, and ensure that critical health and contact information about the child is collected from the parents at that time. Wherever possible, the discussion about the project and consent will happen with parents/guardians and young person together, in an age-appropriate manner, and in the participant’s home. However, this may not always be possible. If the parent/carer does not consent for their child to participate in Yawardani Jan-ga, the child will not be able to enrol in the program.
***2*. *Primary participants aged 16 years and over referred to the program***: Verbal consent will be always be obtained from the young person and written consent will be obtained from the referrer. The parent/carer will be informed about the young person’s participation.
  Once Yawardani Jan-ga receives a referral from a local organisation, the principal researcher or a delegated Aboriginal team member will arrange to visit the young person to clearly explain the research by going through the Participant Information Sheet in an age-appropriate manner. The principal researcher or team member will check the young person understands the information provided and ask any questions the young person may have. The young person will then be invited to provide verbal consent to participate in the program.
***3*. *Opting out*:** Participants of any age can opt out of the program at any point without fear of repercussions or limiting further opportunities for contact throughout the program’s life. This will be emphasised during the consent process.
***4*. *Media consent***: Permission to take photos/video specifically for media or publication purposes (not for purposes of client-file/assessment) will be sought from parents/carers and participants with a separate ‘media’ consent form. Participants who do not sign the media consent can still participate in Yawardani Jan-ga.

***Consent process for secondary participants:***

Staff employed by local organisations interested in referring young people to Yawardani Jan-ga will be requested to read a Participant Information Sheet outlining their role during EAL program and in the evaluation of the program. Referrers will be asked to provide written consent upon referring a young person to the program. As described above, parents/ carers will be asked to provide written consent to the enrolment of their children into the EAL program. By consenting to their child’s participation in the EAL program, they are also consenting to have regular contact with Yawardani Jan-ga staff to ensure their child’s wellbeing is being supported.

During the EAL program, referrers and parents/carers will be asked to have regular check-ins to provide an update on how the young person is tracking, what effect the program is having, and if anything has happened that may affect the young person’s participation in the program. This information is confidential and will be analysed to give a thorough understanding of the gradual impact of Yawardani Jan-ga on the health and wellbeing of the participant and their families. In addition, referrers will be requested to share their experiences referring to the program, their opinion about the perceived impact of the program on young people, and provide feedback or suggestions about how the program could be improved or better address the community’s needs. These evaluation interviews will be de-identified to be identifiable only by profession (e.g., youth worker, psychologist etc).

#### Data collection schedule

Please refer to S3 Fig, for an overview of the open-door participant flow and data collection schedule and Table 1 below for an overview of the data collected.

**Table 1:** Nature of the data collected at each process.

Timing	Process	Data Collected in Client file
**Referral**	Young Person referred to Yawardani Jan-ga by local organisation/service (after discussion with parent/carer)	Person-centred referral (presenting) concerns • Name of organisation • Name and position of person referring. • Contact details of referrer • Location of referrer • Participant name • DOB • Address/ contact details • Aboriginal (Binary item) • Gender • Are parents aware of referral • Statement of primary concern: (Examples: incident details, history of violence, poor impulse control, non-compliance) • High-risk behaviours (Examples: drug/alcohol intake, self-harm/suicide attempts, overt behaviour) • Any additional information (Examples: school completion, contact with police, incarceration, out-of-home care, trauma history)
**Consent**	Participant / Parent consent and Participant health checklist	• Any medical condition that may impact participation in EAL. If yes, details/management (e.g., medication, therapy) • Any allergies to animal hair or asthma. If yes, details/management (e.g., medication, therapy) • Any allergies to medicines. If yes, details/management (e.g., medication, therapy) • Any other information that you think the program should know when interacting with the young person (e.g., Triggers etc.)
**Pre-intervention check**	Participant scheduled for initial EAL session	At each EAL session• Date • Session type • EAL themes/horse wisdom discussed • Photo sequences and/or video footage taken during the EAL session • Voice recorded/written session notes
**Mid-intervention check**	Parent/carer and referrer check in @ 5-sessions. Seek feedback regarding whether and how participants apply learnings across diverse contexts (e.g., school and home). They will also help practitioners determine whether the program requires refinement.	Observed changes in referral (presenting) concerns• How’s participant going? • Is there anything we should be aware off (e.g., home, family, friendships?) that may be affecting the participant? • Have you noticed any change in participant since he/she/they started the program?
**Completion of recommended Dose of 10 sessions**	Parent / carer and referrer check in @ 10-sessions. Participant has attended the recommended dose of 10 SA	Attendance data assessed in relation to referral concerns:• Number of sessions attended • Number of missed sessions + reason • Number of times each session theme is discussed • Number of times each horse worked with young person
**Completion of check after recommended dose of 10 sessions**	Parent / carer and referrer check in @ completion of 10-session. **(Opportunity for re-referral)**	Observed changes in referral (presenting) concerns.• Before the program, what was the young person like? How long had the young person been that way? Have you tried anything to help the young person? If yes, what? • When the young person started the program, what happened? Was there any change? How did you know? What did you notice? • How are things now? Has anything changed?
**3-month post-completion of 10 sessions**	Parent/carer and referrer check in @ 3-months post 10 SA. **(Opportunity for re-referral)**	Observed changes in referral (presenting) concerns
**6-month post-completion of 10 sessions**	Parent/carer and referrer check in @ 6-months post 10 SA. **(Opportunity for re-referral)**	Observed changes in referral (presenting) concerns
**12-month post-completion of 10 sessions**	Parent/carer and referrer check in @ 12-months post 10 SA **(Opportunity for re-referral)**	Observed changes in referral (presenting) concerns

- ***Objective 3*: *Evaluate the implementation and impact of the Yawardani Jan-ga EAL project on SEWB and life skills and plan for its sustainability if found effective*.**

Using a Participatory Research Approach, Yawardani Jan-ga will be qualitatively led in its evaluation and instrument development activities [102,128,129]. Inherent in the Participatory Research Approach is that participants are not simply considered subjects from whom data are collected; the focus is not on collecting clearly defined outcomes or outputs. Rather, participants, their families, and the community are considered active research agents in the research process who possess valuable knowledge and experiences to offer Yawardani Jan-ga on the expression, experience, manifestations and consequences of poor SEWB and life skills in the communities in which they live [102,128,129].

Qualitative methods will be used to understand how participants perceive, feel, process, and create meaning from their experience in the program. Methods employed will include observation, interviews, focus groups, drawings or paintings, and methods that use technology to capture experiences, such as photography, video, and voice recording. This ensures that data collection methods are appropriate for community involvement and engagement for Aboriginal children and young people who may not respond to talk-based data collection. The contextual units of analysis will include the Yawardani Jan-ga EAL program, participants, and the dyadic horse-human interaction. Data will be gathered through multiple and diverse sources, including the client file; interviews with participants, parents/carers, and referrers; minutes and/or transcripts from meetings and consultations with different community groups (Advisory and Steering groups); project documentation; and community information sessions.

Qualitative data analysis will follow the descriptive phenomenology method of inquiry (130). In this context, Colaizzi’s phenomenological approach will be used to provide knowledge about aspects of a young person’s world that cannot be accessed by observation alone, and it, therefore, has implications for understanding SEWB as experienced by young Aboriginal people [103,130,131]. While analysing qualitative data from the initial cohorts of participants, an inductive approach to coding will be adopted to remain open to unexpected insights and themes. In subsequent cohorts, a mixture of inductive and deductive approaches to coding will be used, and themes will be compared across cohorts in an iterative process. At least two Aboriginal research team members will undertake coding and thematic analysis. Congruence and agreement of coding and thematic analysis will be checked using intra-rater and inter-rater reliability indices [132]. We will follow the Consolidated criteria for reporting qualitative research (COREQ) checklist for interviews and focus groups to report the findings of the research [133] (See Supplementary Section, ST2: COREQ 32-item checklist). Qualitative data will be used to:

- Evaluate whether program activities are implemented as intended (process/implementation evaluation).
- Evaluate the impact of the Yawardani Jan-ga EAL program by dose (i.e. how much and which sections of the program) on individual participants from different perspectives (e.g., self, parent/carer, referrer) (Impact evaluation).
- Guide the development and/or adaptation of two instruments that accurately capture important SEWB skills in Aboriginal children and young people.

**Process/Implementation Evaluation:** Gaining a comprehensive understanding of how the Yawarandi Jan-ga intervention is operationalised in the field will allow for the continuous improvement of EAL intervention and inform the development of broader programs seeking to promote the SEWB of Aboriginal youth and enable sustainability. To evaluate the implementation of Yawarandi Jan-ga EAL, assumptions will be developed describing how the intervention works (using a logic model) and how these informed the selection of research questions and methods [134]. A process evaluation framework developed for evaluating and translating evidence into real-world settings and successfully used with First Nations youth will be used [135]. Process evaluation of the EAL intervention will measure the following:

- Readiness of EAL practitioners in implementing the intervention (elaborated in the data sources section below).
- Fidelity and quality of implementation (e.g., What is implemented and how? To what extent was the EAL intervention delivered in accordance with the EAL Practitioner manual?).
- Mechanisms of change (e.g., How does the delivered intervention produce change?).
- Context (e.g., How does context affect implementation and outcomes?. Context includes: *Geographic location of implementation* (Broome, Halls Creek, Derby) and its implementation logistics, including transportation, infrastructure (presence of a dedicated community liaison person and driver), availability of services and support in each location, and environmental conditions; *Community and Social Context*: We will explore the impact of community dynamics, social networks, and local leadership at each site on recruitment, uptake and implementation.
- Barriers and enablers across context (Socio-political environment), recruitment (Approaches used to attract participants) and implementation (What was the acceptability and uptake of EAL? and of the project as a whole? How well did the project link with referring in and referring out services?).
- Reach (The proportion of the intended target audience who participated in the intervention) and dose (the number and which of EAL sessions were delivered).
- What improvements and future recommendations have been made by participants, parents/ carers, referring agencies, EAL practitioners, and stakeholders regarding how the project/ EAL is run? What were the barriers and enablers to success (i.e., the referral process working, young people staying engaged in EAL)?

#### Data sources to inform process/implementation evaluation

Because the effectiveness of the EAL intervention rests primarily on the quality of the training and mentoring received by EAL practitioners, the quality of the EAL intervention will be assessed via three components: Training of practitioners, supportiveness of post-training supervision and mentoring, and delivery of EAL-Intervention. These elements will be captured via various sources, including video footage of EAL practitioner training, notes from supervisor-observed sessions, and transcripts of individual and group reviews of video-recorded EAL sessions. Evaluation interviews with EAL practitioners will be conducted in six-monthly cycles to provide their perspectives on the continuous training opportunities and support they receive to become more confident in their EAL practice.

We will use a multi-modal approach to understand EAL practitioners’ readiness to implement the intervention comprehensively. This multi-dimensional approach ensures that all aspects of readiness—knowledge, skills, attitudes, and perceived preparedness—are thoroughly assessed, providing a solid foundation for successful intervention implementation. This includes:

- *Post-Implementation open-ended questions*: We will ask open-ended questions about their experiences with training, perceived challenges, and confidence in implementing the intervention. Examples of questions: "How prepared do you feel to implement the intervention after the training?"; "What aspects of the training were most helpful in preparing you to work with participants?"; "What challenges do you anticipate in implementing the intervention?
- *Training Observations*: Observe practitioners during training sessions to evaluate their engagement, understanding, and application of the intervention principles
- *Ongoing Supervision*: Implement regular supervisory sessions to provide ongoing support and feedback to practitioners, and Supervisors can assess practitioners’ readiness and provide additional training or resources as needed.
- *Reflective Journals*: Encourage practitioners to maintain reflective journals throughout the training and initial implementation phases and Ask them to document their learning experiences, perceived readiness, challenges, and reflections on their progress.

*In-person interviews with each stakeholder group* will be conducted to explore the barriers and enablers to engaging with the Yawarandi program. Questions will be framed depending on each stakeholder’s role. For example, as a consequence of feeder schools and organisations’ engagement with Yawarandi Jan-ga–the following will be explored: (i) School principal’s/referrer’s perception of their ability to access the program and utilise the referral process and markers as part of mental health response for Aboriginal young people; (ii) The school/ referral organisation’s beliefs about capabilities, motivation, opportunities and skills to refer students to Yawarandi Jan-ga; (iii) The school/ referral organisation’s perceptions of the appropriateness of the Yawarandi Jan-ga referral process and impact of the results of Yawardani Jan-ga on participants; and (iv) Whether additional training or opportunities are needed to improve and maintain student engagement with the program.

*Other data sources* will include minutes and/or transcripts from meetings and consultations with different community groups (Steering / Advisory Committee), planning documents, budgets, promotional materials, presentations, and funding proposals. All qualitative data, including post-session interview reflections with participants, video footage of sessions, EAL practitioner notes after each session and photographs of each session, will be triangulated to provide a comprehensive process evaluation.

**Impact evaluation:** Data will be captured in a comprehensive database and analysed to determine the impact of the intervention. The analysis will consider questions such as:

- Did EAL improve the SEWB and life skills of participants?
  ○ Domains of interest include self-regulation, self-awareness, confidence, and prosocial behaviours.
- What changes did participants notice in themselves?
- What changes did carers and/ or referring agency staff notice?
- For whom does EAL seem to work best, and under what conditions?
- How much EAL is needed to change SEWB outcomes (Dose, fidelity and intensity) using a mixed-method approach.
  ○ Both dose and fidelity will be measured by tracking the number of sessions completed or conducted and the proportion of each session that adhered to the recommended content. This will involve observations of the practitioner and reports, resulting in responses that may include both quantitative and qualitative components
- Is the content and sequence of EAL sessions meeting the needs of youth in the Kimberley region (adding, removing, or re-ordering sessions)?

Variables of interest will include referral concerns; individual baseline data collected by local services (e.g. police contacts, school attendance, and mental health contacts); EAL-attendance (e.g. dates of each session, number of sessions, frequency) and EAL-progress (e.g. notes, photos, videos collected during EAL-sessions); any variations in delivery (e.g. reason for any temporary exit from the intervention, change in session order); notes taken during check-ins with participants, parent/carer/significant other, and referrers (at 5-session attendance, 10-session attendance (10 SA), 3-months after completion of 10 SA, 6-month after completion of 10 SA and 12-month after completion of 10 SA). In addition, the number of participants enrolled during the 30-month enrolment phase in each site will be included in the Impact analyses (S1 Fig Timeline of Yawardani Jan-ga Project). The timeline also allows for the staggered collection and analysis of follow-up data for each participant. The timelines of Objectives 2 and 3 are somewhat overlapping; once the program is implemented at one site, it may then be rolled out to another. Consequently, the evaluation process begins as soon as the training and program are initiated and continues throughout the implementation. As detailed previously, descriptive phenomenology will be used [103,130,131]. We will follow the Consolidated criteria for reporting qualitative research (COREQ) checklist for interviews and focus groups to report the findings of the research [136] (See Supplementary Section, ST2: COREQ a 32-item checklist).

#### Primary efficacy endpoint

*Improved community engagement indicators of SEWB and life skills*: The following open-ended questions will be asked to referrers to allow respondents to express their thoughts and feelings freely.

1. Have you noticed any changes or differences in him/her lately / since we last spoke? (refer to referral form -statement of primary concern):
2. How has the [name_young person] been getting on with friends/family since we last spoke?
3. Has anything happened recently that may have affected him/her? Has anything changed in the young person’s family/friendships/community since we last spoke?

Meaningful change will be captured phenomenologically, using culturally appropriate and sensitive approaches (in this case, yarning). We propose using standard thematic analysis methods and comparing across time points? [102,132].

Additional information shared by participants, their parents/ carers and referring agencies about changes they have experienced in engagement as per data collected from services will also be captured including (information may be qualitative or quantitative).

- Relational Health: Family and peer (observed and self-reported improvements in quality of relationships, number of relationships, less conflict)
- Physical Health (e.g., exercise, activity, nutrition)
- Education: School attendance, class participation, school completion
- Justice: Contact with police, arrest
- Risky behaviours: Drug and alcohol use, self-harm, and other risky behaviours

#### Secondary efficacy endpoints

There is a lack of culturally relevant and validated tools to capture the progressive SEWB changes anticipated from alternative therapies such as EAL [66]. Hence, Yawardani Jan-ga proposes to develop and validate an instrument that captures SEWB. This instrument has emerged from identifying key SEWB themes from voice recordings capturing participants’ learnings from EAL sessions and in consultation with Aboriginal young people who participated in the program in 2021–2022. Focus groups with the Steering, Advisory and Youth feedback groups will be undertaken in 2024 to develop an appropriate ’glossary’ of the participants’ vocabulary and behavioural manifestations around SEWB. The exact type of instrument to be developed (qualitative or quantitative) will be determined in consultation with the community, particularly Aboriginal young people. The instrument will also seek to establish cultural and content validity by seeking advice and guidance from the Scientific Steering Committee.

Data from this project will also be used to develop instruments to systematically capture horse-human interactions to build on previous equine psychotherapy research. The first step in developing an EAL-specific instrument will be to identify critical observable interactions of interest between the client and the horse through the analyses of photo and video footage collected during EAL sessions. The next step will be to test a coding scheme to quantify these interactions. Data will be retrospectively assessed according to the Socio-Emotional Health Analysis of Photographic Expressions (SHAPE) coding method that quantifies and rates equine-human dyadic interactions [137]. The measure will likely have both quantitative (number of observations) and qualitative (explanatory) observations. These outcomes will be triangulated with behavioural, educational, and social outcomes reported by parents/carers, teachers, and referrers collected after sessions five and 10. Artificial intelligence and machine learning systems will be explored to bring rigour and efficiency to the SHAPE coding system.

### Sustainability planning of the Yawardani Jan-ga project should it be found to be effective

If the intervention is successful, the last year of the project will focus on transitioning Yawardani Jan-ga from a research project to a sustainable, routine SEWB service. Success will be determined using multi-modal data collection approaches that measure: (i) Success in attending the initial dose of 10 sessions using attendance records; (ii) The proportion of referrers who report the intervention was beneficial for the participants at 3, 6, and 12 months; and (iii) The combination of subjective reports (collected from referrers at 3, 6, and 12 months) and improvements in the proposed equine-human interaction measure (that is being developed retrospectively using data), retention rates, engagement levels, or qualitative feedback on participants’ experiences.

During this phase, the focus will be on consolidating service-related mechanisms trialled throughout the project (e.g., contracts, agreements, intervention provision) to enable EAL to be provided as part of routine SEWB programs within health, education and other non-health related services across the Kimberley. Yawardani Jan-ga will seek full-service integration through negotiating service agreements in each site with project partners (e.g., police, schools, Aboriginal Medical Services), including training and employment paths for current and future EAL-practitioners and securing a host organisation to manage the maintenance of horses and facilities.

## Translation/Dissemination/Publication

Dissemination of progress and outcomes will occur throughout the life of the project at multiple points through various channels:

- At the participant level, verbal and visual (photos and video) feedback to participants and caregivers will be used so that they can create meaning from their experience in the program.
- At the community level, study results will be disseminated through community engagement events, including local meetings involving participants, their families, and the wider community. In addition, the program will work directly with community members and groups to analyse and interpret data to guide the future direction of Yawardani Jan-ga. This approach will embed an Indigenous worldview, perspectives, and values at the core of Yawardani Jan-ga feedback to communities, ensuring the information disseminated is culturally appropriate and accessible on each site. For example, results and interpretation will be discussed with local groups before community meetings, including:
  ○ Steering Committee comprises representatives from local Aboriginal medical services, youth and mental health services, and traditional owners.
  ○ Youth Feedback group comprises current and ex-Yawardani Jan-ga participants.
  ○ Yawardani Jan-ga’s CIs and AIs sit on multiple State and National Boards. This project will, therefore, have high-profile platforms to disseminate study results and share its experiences.
  ○ We also have an active Elder Advisory Group.
- At a service and policy level, significant engagement has already begun with health and mental health services, schools, justice, police, youth services and others (including the West Kimberley District Leadership Group) about the program and potential benefits. Services and agencies are supportive and keen to participate by referring young people to the program. Clear synergistic benefits and future pathways to sustainability have already been identified.
- For the Scientific community, manuscripts will be prepared for publication in peer-reviewed journals at different time points throughout the project.
- To support community engagement and awareness, an interactive Facebook page and project webpage will be developed.

**Advancing indigenous research processes:** Through Aboriginal leadership, community engagement, capacity building, and service partnership, Yawardani Jan-ga will advance the development, articulation, and adoption of Indigenous research processes that capture cultural protocols and community dynamics and align with Aboriginal worldviews. Our approach has the potential to create innovation for how future programs and services aimed at enhancing the SEWB of Aboriginal people are developed, delivered, evaluated, and translated into practice. What works for Aboriginal children and young people is currently not being developed or evaluated using culturally secure measurement approaches. Processes that bring family, community, culture, and country to the centre of services and respectfully implement and measure outcomes in an Aboriginal context need to be further explored and validated in ‘*true partnership with Aboriginal communities’*. This project will also improve our understanding and appreciation of how SEWB is expressed by Aboriginal children and young people and, in doing so, inform the future development and implementation of successful culturally secure interventions and measurement approaches.

## Supporting information

S1 Fig Timeline of Yawardani Jan-ga project. (TIF)

S2 Fig Subcomponents of the Yawardani Jan-ga project. (TIF)

S3 Fig The open-door flow of participants and data collection schedule. (TIF)

S1 File Overview of Yawardani Jan-ga EAL sessions. (DOCX)

S2 File Consolidated criteria for reporting qualitative research (COREQ): A 32-item checklist for interviews and focus groups. (PDF)

S3 File Yawardani Jan-ga information sheet and consent forms. (PDF)
