# Supplementary figures and images for: Protocol to implement and evaluate a culturally secure, strength-based, equine-assisted learning program, "Yawardani Jan-ga" (horses helping), to support the social and emotional wellbeing of Australian aboriginal children and young people
Source: PLoS One. 2024 Dec 30;19(12):e0312389. doi: 10.1371/journal.pone.0312389 (PMC11684595; doi:10.1371/journal.pone.0312389)

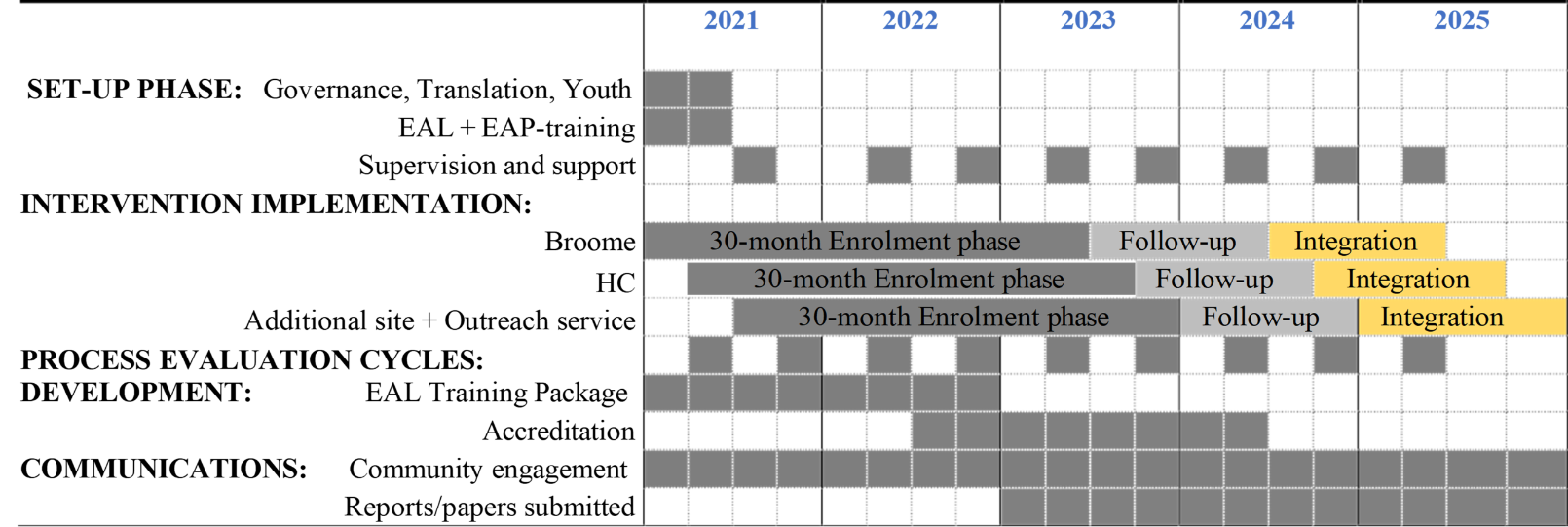

Supplement: S1 Fig — (TIF) [file pone.0312389.s001.tif]

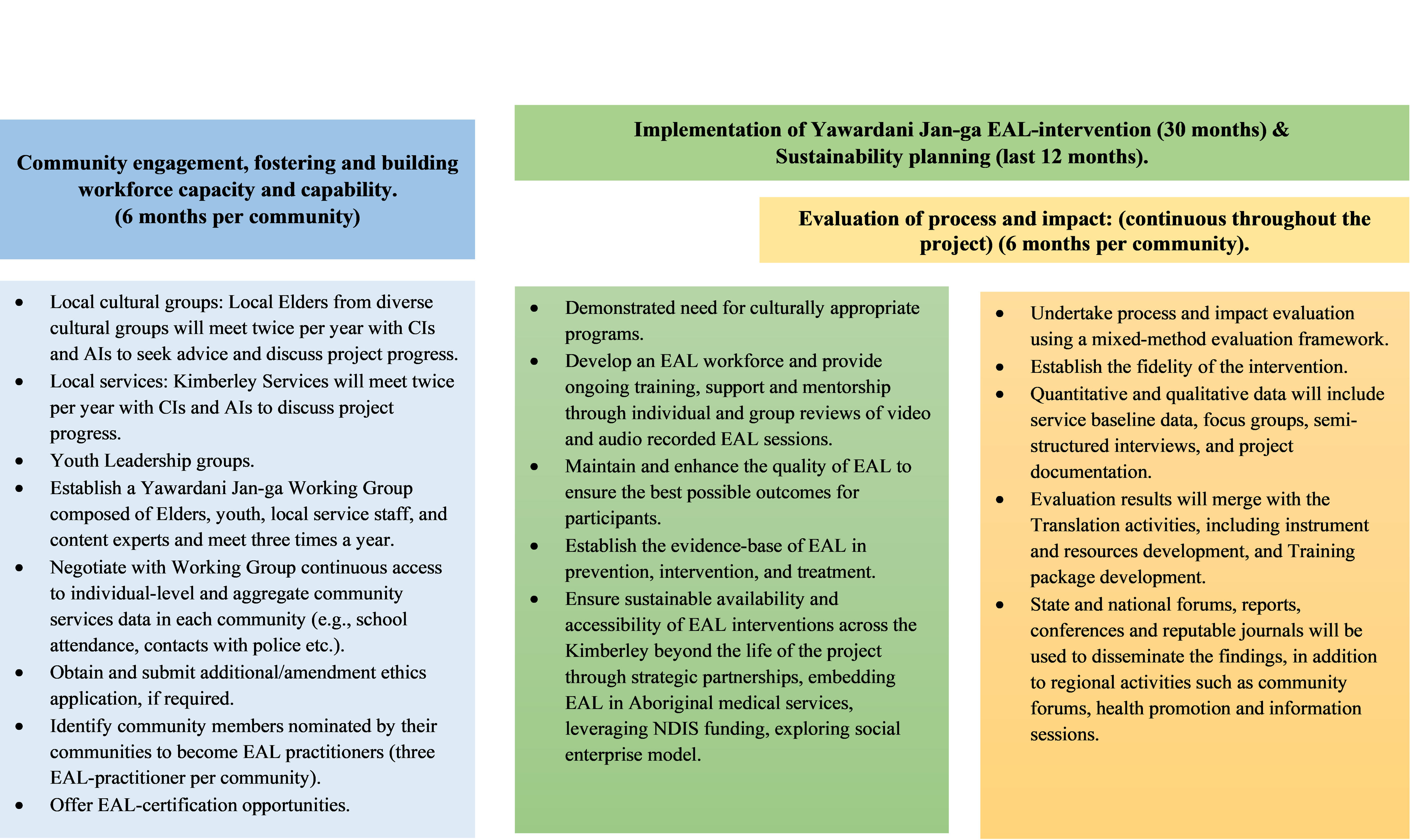

Supplement: S2 Fig — (TIF) [file pone.0312389.s002.tif]

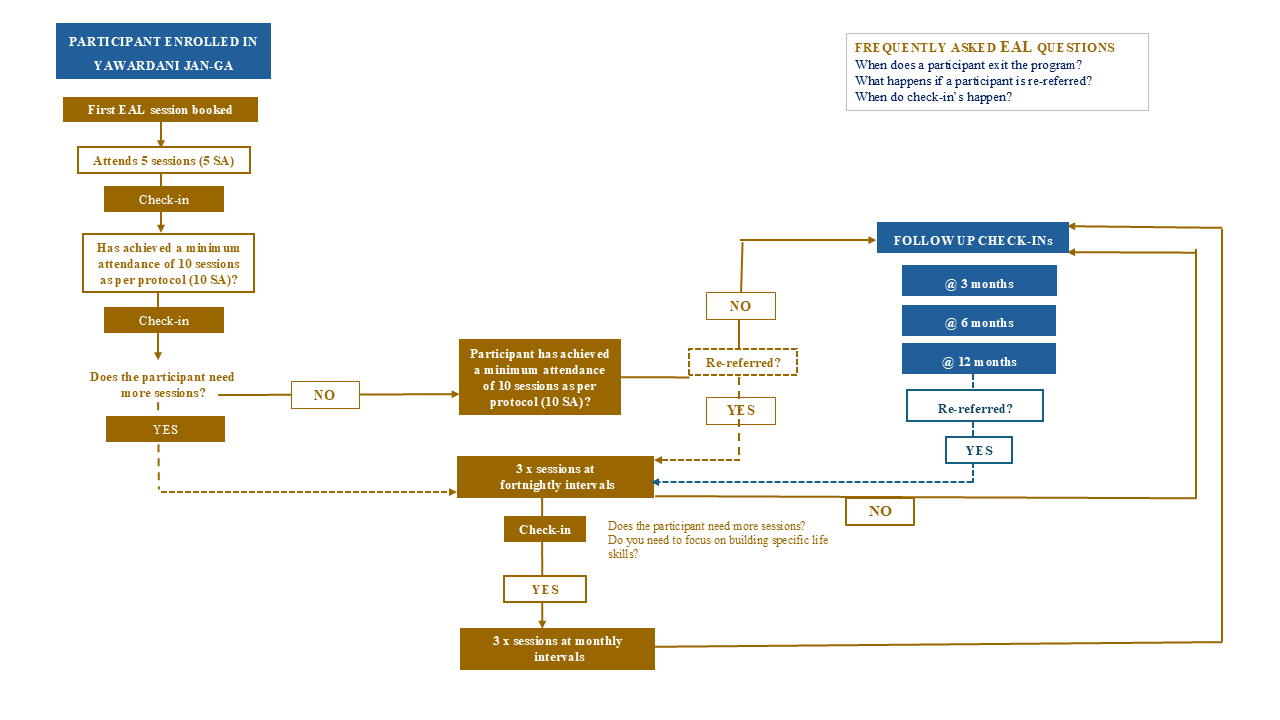

Supplement: S3 Fig — (TIF) [file pone.0312389.s003.tif]
