# Supplementary material for: Protocol to implement and evaluate a culturally secure, strength-based, equine-assisted learning program, "Yawardani Jan-ga" (horses helping), to support the social and emotional wellbeing of Australian aboriginal children and young people
Source: PLoS One. 2024 Dec 30;19(12):e0312389. doi: 10.1371/journal.pone.0312389 (PMC11684595; doi:10.1371/journal.pone.0312389)
Supplement: S1 File — (DOCX) [file pone.0312389.s004.docx]

**Table 1:** Overview of Yawardani Jan-ga EAL sessions

| **Session #** | **Theme** | **Definition of Theme** | **Sample Activity** |
| --- | --- | --- | --- |
| 1. | Noticing | Noticing when you are becoming activated and how  to regulate your nervous system using simple tools | Observe horse behaviour and herd dynamic |
| 2. | Awareness | Awareness will give you a choice. Awareness of Self,  including thoughts, feelings and sensations, awareness of others and awareness of the environment. How to become present, aware, and grounded. Noticing body language. | Touch/groom the horse and observe how horses demonstrate self-regulation and co-regulation (support each other) |
| 3. | Boundaries | Understanding and becoming respectful of personal  space, touch, and verbal boundaries. To discover what is OK and NOT OK. How to communicate these clearly | Lead horse using direct (voice and touch) and observe horse response to participant approach, voice, and touch |
| 4. | Healthy Relationships | The ingredients are necessary to engage and develop.  healthy relationships. Noticing and understanding self and noticing others, give and take, mutuality. Rupture and repair work. | Move horse in yard using direct (voice and touch) and indirect (energetic) connection and interpret horse and own body language |
| 5. | Helpful thoughts and behaviours | Becoming aware of self-talk, beliefs and where they  come from. How thoughts affect your energy, body language and actions. Noticing helpful or unhelpful thinking. | Move horse in yard using indirect (energetic) connection only and relate horse behaviour to current thoughts |
| 6. | Feelings as natural | Understand that feelings are information, all feelings are  welcome, and learn how to express them choice-fully and healthily. Become aware of the early sensations so that you can act earlier. Surf the urge. | Move horse through an obstacle course using direct (voice and touch) and indirect (energetic) connection |
| 7. | Facing life challenges | How to deal with life and the challenges it  throws at us. | Build an obstacle course and lead horse at liberty through it (obstacle represents obstacles in life) / mounted session |
| 8. | Paired session to consolidate skills/learnings |  | Working in pairs to build an obstacle course and lead horse through it. Emphasis on working together to achieve a common goal |
| 9. |  |  |  |
| 10. | Small-group session to consolidate skills/learnings |  | Working in a small group (n = 4) to build an obstacle course and lead a horse through it. Emphasis on working with others to achieve a common goal |

*Each activity meets three key elements: (1) activities are action and skill focused on building participant sense of efficacy and capability; (2) 'mindful disengagement' (e.g., ability to step back, observe and label feelings); and (3) develops and reinforces self-regulation skills.
