# Supplementary material for: Protocol to implement and evaluate a culturally secure, strength-based, equine-assisted learning program, "Yawardani Jan-ga" (horses helping), to support the social and emotional wellbeing of Australian aboriginal children and young people
Source: PLoS One. 2024 Dec 30;19(12):e0312389. doi: 10.1371/journal.pone.0312389 (PMC11684595; doi:10.1371/journal.pone.0312389)
Supplement: S3 File — (PDF) [file pone.0312389.s006.pdf]

PRIVATE-CONFIDENTIAL

Yawardani Jan-ga

Parent/Carer Consent Form

By signing this consent form I declare that I accept that I have understood the following:

- I understand this project is trying to find out what benefits EAL has on Aboriginal children and young people.
- I understand that my child has to leave the school grounds to take part in Yawardani Jan-ga.
- I understand the risks of allowing my child / the child in my care to take part in this project.
- I agree for my child to be part of the project and understand he/she can say 'no' or 'stop' at any time.
- I understand that the purpose of 'check-ins' with the organisation that referred my child to Yawardani Jan-ga are for tracking my child's progress.
- I agree to have 'check-ins' with Yawardani Jan-ga staff about how my child is going.
- I understand that my child's school attendance will be requested by researchers to see if the program changes their school attendance.
- I understand that information collected about my child will be combined with the information collected from all other children enrolled in Yawardani Jan-ga to understand the overall impact of the program, and how to improve the program for other young people and families.
- I understand that general feedback about the program given to community or research reports will not reveal my child's name or personal information.
- That information about my child will be stored confidentially and will be analysed by Yawardani Jan-ga researchers to assess the impact of program on my child and improve the program. I have been given enough information and the opportunity to ask questions.
- I have been given a copy of the Information Sheet to take home.

PRIVATE-CONFIDENTIAL

I ____ have read and understood the "Information for
(Parent/Caregiver name)

Parents and Carers" and give permission for ____ to:
(Child's name)

I give permission for my child to participate in Yawardani Jan-ga.	Yes ☐	No ☐
If the school Principal agrees, I give permission for my child to leave school to take part in Yawardani Jan-ga.	Yes ☐	No ☐
I give permission for notes, photos and /or video and voice to be recorded during Yawardani Jan-ga to track my child's progress.	Yes ☐	No ☐
I understand that general feedback about the program given to the community or research reports will not reveal my child's name or personal information.	Yes ☐	No ☐
I agree to have 'check-ins' with Yawardani Jan-ga staff to help improve the program.	Yes ☐	No ☐
I give permission for myself and my child to be contacted in the future.	Yes ☐	No ☐
I give permission for the school to give the Yawardani Jan-ga researchers my child's attendance and absence rates (authorised and unauthorised) for the semester before, during and the semester after your child stops attending the program.	Yes ☐	No ☐
I give permission for all information collected about my child by Yawardani Jan-ga staff to be used to work out the overall impact of the program, and to improve the program.	Yes ☐	No ☐
I give permission for the information collected about my child to be used in presentations to the community or included in research reports as long as my child cannot be identified.	Yes ☐	No ☐
I give permission for the following arrangements for my child to get to and from the program.<br>• School/Organisation to provide a drop off/pick up service.<br>• Yawardani Jan-ga program to provide a drop off/pick up service.<br>This arrangement will in place for the duration of the child's participation in the Research Program.	Yes ☐	No ☐

Signature: ____ Today's Date: ______________________________

Thank you for taking part in Yawardani Jan-ga.

PRIVATE-CONFIDENTIAL

Yawardani Jan-ga

Parent/Carer Check list

Date completed:	
Name of person(s) completing check list:	

**CONTACT INFORMATION**		
Name of child or young person:		*Weight (kg):*
School Name:	Grade:	Form class / classroom teacher:
Emergency contact name and phone number:		relationship to the young person
Alternate contact person name and phone number:		relationship to the young person

**HEALTH HISTORY**<br>Please indicate any medical condition that may impact participation in the Yawardani Jan-ga program	
	**Details / Management (e.g. medication, therapy)**
Does your child have any allergies:	Tick all that apply:<br>☐ To animal hair *(details):*<br>☐ To any medicines (details):<br>☐ Asthma *(details):*<br>☐ Other *(details):*
Is your child taking any medications?<br>*in the case on an emergency, incident or injury, this information will help us inform first aid responders.*	Please tick:<br>☐ No<br>☐ Yes *(details):*
Is there any other information about your child you think we should know?	

PRIVATE-CONFIDENTIAL

Yawardani Jan-ga
Information for Parents and Carers

Welcome

The Ngangk Yira Institute for Change, Murdoch University is running the Yawardani Jan-ga (Horses doing healing) Equine Assisted Learning (EAL) research program in Broome, Derby, Halls Creek and other localities in the Kimberley region.

We would like to ask permission for your child to be part of the Yawardani Jan-ga EAL Program. It is important to know about this project and what it involves before saying "Yes" or "No" to your child taking part.

What is the Yawardani Jan-ga research program about?

Yawardani Jan-ga means 'horses doing helping' in Yawuru language.

In this research, we are seeing what benefits EAL has for young people.

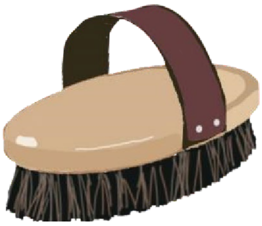

In Yawardani Jan-ga horses are the teachers. During the program, your child will work with, or around horses on the ground doing activities like grooming the horse or leading the horse through an obstacle course following the instructions from a trained EAL Practitioner. A mounted session may be offered to your child if the EAL Practitioner thinks it is safe and appropriate to do so.

These activities with the horse help show young people life skills, such as how to recognise and understand their feelings, coping and emotional regulation strategies, friendship making skills, and helpful self-talk. These life skills help build healthy relationships with others, as well as demonstrate a different way of responding to life stresses. This is called "Experiential Learning" and is one of the only ways we can develop life-long learning and become more mindful.

How does my child access the program?

As a parent/carer you must provide consent for your child to participate in the program and consent for how they will get to and from the program.

A Referral Form must be submitted to Yawardani Jan-ga from a local service provider. This means your child's teacher, youth worker, police, or other can complete a Referral Form with your permission.

The Referral Form requests information about your child's challenges, strengths, and supports. This information will be used to plan the program for your child and track your child's progress during the program.

What does my child have to do?

For Yawardani Jan-ga, young people do not need any experience with horses.

Most children will attend Yawardani Jan-ga for one session a week for one school term (about 10 weeks). Depending on what your child needs help with, they may be enrolled longer or even on an on-going basis.

Sessions last 45 minutes to 1-hour and are held during school hours (8am – 2:15pm). Yawardani Jan-ga program staff will talk to you and the school Principal to arrange pick-up and drop-off of your child from school or home.

Each session begins with a horse-safety briefing and check-in around social and emotional wellbeing.

Every session will be different and planning between the EAL Practitioner and Professor Juli Coffin will occur before and after every session. The focus is on the experience that the young person has, and the feedback provided from the horse. All sessions are guided by a local Aboriginal trained EAL Practitioner.

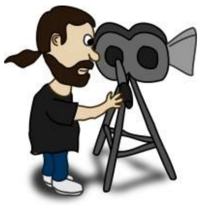

During the sessions, the EAL Practitioner may take notes to document progress and make plans for the next session. Sometimes, we will ask if it is ok for us to take photos, videos, or voice recordings. This is one way we track the progress your child is making in the program. Each session, your child can say 'yes' or 'no' to the photos, video, or voice recordings without missing out on the horse experience.

What if my child doesn't want to do one of the activities in the session?

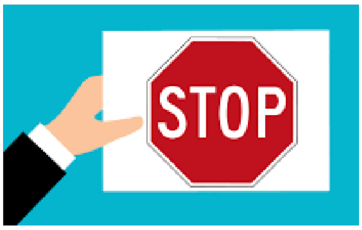

If we start a session and your child decides that he/she wants to stop, they can stop the session by simply saying they wish to stop.

In the Yawardani Jan-ga program, your child is in-charge of their time and can say 'yes' or 'no' to yarns, video recordings, photos and activities. This will be encouraged by the EAL practitioner to build self-awareness and 'choice'. No one will mind if they want to stop.

Are there any risks to my child?

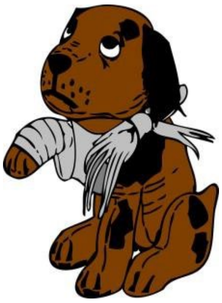

Every session begins with safety instructions and a check-in around their social and emotional wellbeing.

Horses are big animals and can sometimes act in sudden and unpredictable (changeable) ways, especially if frightened or hurt. The horses are in a natural state and can move freely and quickly at times. The EAL Practitioner is responsible for monitoring the session and stopping and/or changing the experience if, in their opinion, there are any safety risks to your child.

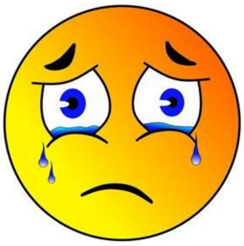

Should your child become upset or need further support, the Yawardani Jan-ga program has support from local youth counselling services, and online/phone access to trained Aboriginal counsellors if an in-person visit was not possible.

All Yawardani Jan-ga program staff and researchers are required to have a Working With Children Check Card and WA Police Clearance.

As a parent/caregiver, what do I have to do?

For your child to be part of this program, you will be required to do two things:

1. You will need to give your consent by signing a Consent Form. If your child currently attends school, there is a section in the Consent Form that seeks your permission for your child to leave the school premises (or location) during the school day. Depending on each school procedures, you might need to speak directly to the school principal about giving your child permission to leave school for one hour

each week. You will also need to give permission for the arrangements about how your child will get to and from the program.

2. We will request to check-in with you mid-way (5-weeks), towards the end (10-weeks), and approximately 6-months after the last EAL session. These check-ins last about 5 minutes and are to see if what your child is learning from the horses is being applied at home. The information collected from you through these check-ins is another way to track the progress your child is making. During these check-ins notes may be taken by the EAL Practitioner. You can say 'no' to these check-ins. It will not stop your child from being in Yawardani Jan-ga.

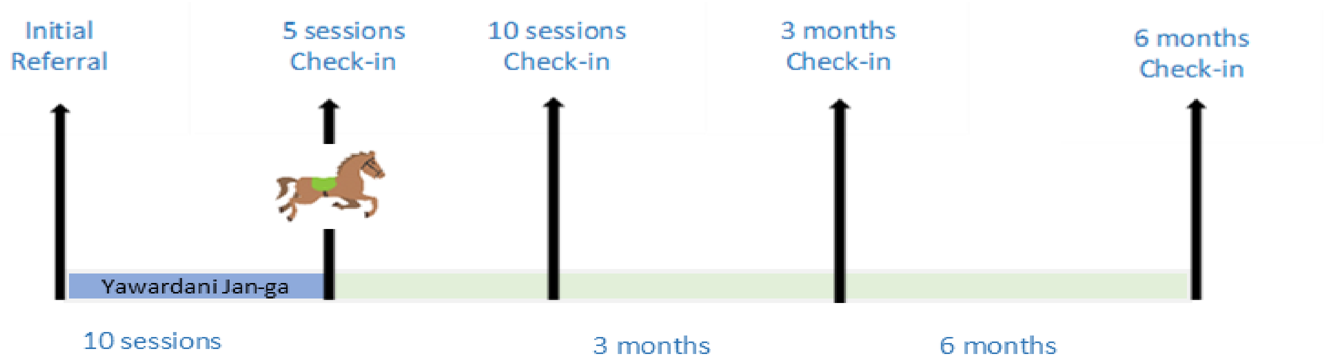

Figure 1: Timing of 'check-ins' with Referrers to track progress in Yawardani Jan-ga

What kind of information do you collect about my child?

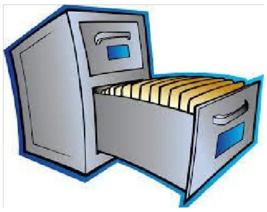

Yawardani Jan-ga will keep a 'Participant-file' on each young person taking part in the program. The Participant-file will contain personal information such as the Referral Form, session notes, photos and videos of child-horse interactions, and feedback from check-ins with the Referrer and the Parents/Carers.

Check-ins with people in regular contact with your child will help us track the progress of each young person, and find out if the skills gained in the EAL-program are being transferred into other settings, like the school, the home, and the wider community. Six months after your child's last session, we will request to speak with you again to see how your child is going, and also to request feedback about the program, and how we can improve it for other families.

In the future, we would also like to check in with you and the organisation that referred your child to Yawardani Jan-ga to help us understand the long-term impact of this program on your child.

Finally, we would like to know if Yawardani Jan-ga encourages kids to go to school. With your permission we will ask the school to provide us with your child's attendance and absence rates (authorised and unauthorised) for the semester before, during, and the semester after your child stops attending the program.

How will information you collect about my child be used?

Information collected about your child will be used to:

- Understand if and how Yawardani Jan-ga is helping your child, in both the short and long term.
- We will also combine the information we collect about your child with information we collect about other young people enrolled in Yawardani Jan-ga. This will help us determine if and how the program has helped Aboriginal young people as a group, and also help us make the program work specifically for the Kimberley.
- We will also combine information we collect about your child with the 'attendance' information collected from your child's school.

How will you keep my child's information private?

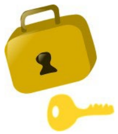

All information stored with Yawardani Jan-ga is **confidential** and is kept under lock and key at the EAL site and on a password protected computer that only the Dr Juli Coffin, the Program Manager, and the EAL practitioners have access to.

Our research team will review the Participant-files independently and confidentially to assess the progress made by each young person according to different people who have contact with your child in different settings (for example, the school, the home etc).

The information we collect about each participant will not be shared with any other services without consent of the parents/carers and the participant themselves, unless:

 the participant becomes involved in a serious criminal matter where others are at risk; or

 we perceive the participant to be at risk of self-harm or harm to others; or

 we are required to by the law.

As required by Murdoch University, all the information we collect about your child will be stored securely for at least 7 years after we publish a report or project completion or until your child turns 25 years of age, whichever is longest. After this time, all information will be destroyed.

Will I receive feedback on my child's progress?

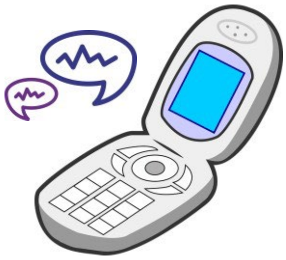

Yes. Contact with parents/carers is necessary, and we will contact you regularly to let you know how your child is going in the program.

Also, general results about the whole program will be presented at community meetings and be published in the form of a report. When general feedback about the program is given to community, no personal information about people involved will be disclosed. This means that the name of your child, the name of the school, and the names of people we spoke to about your child's progress will not be made public.

Is there anything else I should know about this study?

Yes. We would like to ask your permission to contact you and your child **in the future to** catch-up and see how your child is going. For example, we might ask you to tell us what your child is up to, or we might ask your child how they think the program helped them, if at all. Right now, we are only asking for permission to contact you and your child in the future. When we contact you in the future, you can say 'yes' or 'no' to talking to us. It will be completely up to you.

What if I have a complaint?

Approval to conduct this project has been provided by the Western Australian Aboriginal Health Ethics Committee. Any person with concerns or complaints about how we have behaved in your community may make any complaints by contacting the Western Australian Aboriginal Health Ethics Committee (08) 9227 1631 or by emailing ethics@ahcwa.org.

What if I have more questions or want more information?

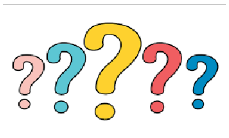

If you require further information or want to ask questions, please contact:

Professor Juli Coffin on 0436 454 422 or yawardani.smb@murdoch.edu.au

Our partners:

PRIVATE-CONFIDENTIAL

Yawardani Jan-ga

Referrer Consent Form

By signing this consent form I declare that I accept to participate and that I have understood the following:

- I understand this project is trying to find out what benefits EAL has on Aboriginal children and young people.
- I understand that I must discuss my intent to refer the young person to Yawardani Jan-ga with the parent(s)/ carer(s) and gain their permission to provide personal information, before completing the Referral Form.
- I understand that the information provided in the Referral Form will be used as baseline from which to track participant progress during Yawardani Jan-ga.
- I understand that I will be contacted by Yawardani Jan-ga staff to have 'check-ins'.
- I understand that 'check-ins' with Yawardani Jan-ga staff will be used to monitor and assess participant's progress in Yawardani Jan-ga.
- I understand that I may be contacted by Yawardani Jan-ga staff to provide feedback about program for evaluation purposes.
- I understand that the information I provide will be combined with information collected from other participants, to evaluate the overall effectiveness of the Yawardani Jan-ga and to adapt the EAL program to the Kimberley.
- I understand I can say 'no' or 'stop' at any time.
- I understand that general feedback about the program given to the public will conceal my identity as well as the identity of participants and schools.
- I have been given enough information and the opportunity to ask questions.
- I have been given a copy of the Information Sheet to take home.

Referrer Name ____ I consent to:

Completing a Referral Form for a child/young person, with their parent/carer consent	Yes ☐	No ☐
• Have 'check-ins' with Yawardani Jan-ga staff for the purposes of monitoring the progress of the young people I refer to the program	Yes ☐	No ☐
• Help evaluate Yawardani Jan-ga (share experiences in referring to the program, any feedback on the program, and its impact on young people)	Yes ☐	No ☐
• I give permission to be contacted in the future about the young person(s) I have referred to Yawardani Jan-ga for the purposes of understanding the long-term impacts of EAL on participants	Yes ☐	No ☐

Signature: ______________________________ Today's Date: ______________________

Thank you for taking part in Yawardani Jan-ga.

PRIVATE-CONFIDENTIAL

Name of organisation: ____

Person referring: ____

Position: _________________________ **Phone:** __________________

Email: ____

Site: Broome / Derby /Halls Creek/Other_________________

(circle one)

Date: _____________________________

Yawardani Jan-ga Referral Form

**PARTICIPANT INFORMATION**	
Participant Name:	
DOB:	Age:
Aboriginal *(circle appropriate)*: Yes No	Gender:
Address:<br><br>Contact number:	
IF APPLICABLE:<br>Do parent(s)/carer(s) agree to be contacted by Yawardani Jan-ga? *(tick appropriate)* Yes No	
**PARENT / CARER INFORMATION**	
Name:	Phone:
Address:	Relationship to child:
Email:	
**Statement of primary concern:** *(Examples: incident details, history of aggression, poor self-regulation, drug/alcohol intake, self-harm/suicide attempts, contact with police, disengagement from school, bullying, recent community or family events)*	

**Potential triggers** *(Examples: past events, not feeling in control, bullying, feeling judged, smells, loud noises)*

**Strengths** *(Examples: interests, communication, family supports, friendships, coping strategies)*
**Any additional information we should know** *(Examples: Health conditions, disability diagnosis, current medications)*
**OFFICE USE ONLY**<br>*(after discussion with parent / guardian / carer / referral service)*
**Recommended frequency and duration of sessions:**<br><br>Type of Format: ______ Group Work ________ Individual Work _______ Family Work
**Treatment goals:**

PRIVATE-CONFIDENTIAL

Yawardani Jan-ga
Information for Referral Services

Welcome

The Ngangk Yira Institute for Change, Murdoch University is running the Yawardani Jan-ga Equine Assisted Learning (EAL) research program in multiple sites across the Kimberley including Broome, Derby and Halls Creek and other localities in the Kimberley region. Through this research we seek to understand what benefits EAL has for young people. It is important that you read and understand the information below to figure out if Yawardani Jan-ga is something that may be suitable for someone you know.

What is the Yawardani Jan-ga research program about?

Yawardani Jan-ga means 'horses helping' in Yawuru language. The aim of the Yawardani Jan-ga project is to support the social, emotional, and spiritual wellbeing of Aboriginal young people through the implementation of culturally secure Aboriginal led and run equine-assisted program.

In Yawardani Jan-ga horses are the teachers. Participants go through a series of interactions with horses under the supervision of a qualified local Kimberley Aboriginal EAL Practitioner. The interactions between participant and horse creates feedback loops which develop essential life skills including emotional regulation, self-awareness coping skills and helpful self-talk. These life skills can help build healthy relationships with others, as well as demonstrate a different way of responding to life stresses.

The 'learning' component in Yawardani Jan-ga arises through repeated observation, practice, and reflections of horse-client interactions, guided by trained local Aboriginal qualified EAL-practitioners. A mounted session may be offered at the discretion of the EAL Practitioner.

Each participant attends weekly one-hour sessions for approximately 10 weeks (one school term). Depending on the young person's referral, they may need to be enrolled longer or even on an on-going basis. Sessions are held during school hours (8am – 2:15pm). Yawardani Jan-ga program staff may be able to provide transport for participants. They will work out transport arrangements with the school Principal and each child's parent/carer.

What does a session look like?

Each session begins with a check-in around horse-safety and social and emotional wellbeing. A range of topics are explored in Yawardani Jan-ga, meaning every session will be different and tailored to suit each young person how they present on the day. This planning is completed between the EAL Practitioner and lead researcher, Professor Juli Coffin, before and after every session.

During the sessions, the EAL Practitioner documents observations and things the child may have said or done. Sometimes we will ask permission to take photos and or videos. These methods are some of the ways in which the program tracks the progress of each student during Yawardani Jan-ga, and also helps plan subsequent sessions.

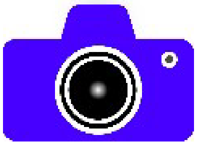

What kind of participants are suitable for the program?

Young Aboriginal people between the ages of 6 and 25 are eligible to be part of the Yawardani Jan-ga program. The program is suitable for all Aboriginal young people, including those with complex social and emotional wellbeing needs, and those demonstrating leadership potential. The program provides prevention, intervention and treatment of issues facing young people.

If a young person is receiving mental health support, it is important they continue to receive mental health support during their enrolment in the Yawardani Jan-ga program.

Are there any risks to taking part in Yawardani Jan-ga?

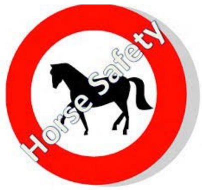

All participants are given a safety briefing every time there is contact with the horses. The horses are in a natural state and can move freely and quickly at times. The EAL Practitioner is responsible for monitoring the session and stopping and/or changing the experience if, in their opinion, there are any safety risks.

Should a young person become upset or need further support, the Yawardani Jan-ga program has support from local youth counselling services and online / phone access to trained Aboriginal counsellors if an in-person visit is not possible. However, EAL does not deepen into emotional psychotherapy and the focus of EAL sessions remains on the young person's interaction with the horse.

How do young people access the program?

If the potential participant is enrolled in a local school, parents/carers must provide consent. In this case, a Referrer *(e.g. teacher, police, doctor, or mental health professional)*, must discuss their intent to complete a Yawardani Jan-ga Referral Form with the parent/carer, and obtain parent/carer permission to share their contact information with Yawardani Jan-ga.

A Yawardani Jan-ga employee will ensure that parent/carer consent is collected before a child is enrolled in the program. Once Parent/Carer consent has been collected, the Referrer must also provide consent and may then submit a completed Referral Form for each young person they want to refer to the program.

If all Yawardani Jan-ga spots are full during a school term, the young person will be put on a wait list and the program will be offered as soon as possible.

As a Referrer, what do I have to do?

We will require you to complete a Yawardani Jan-ga Referral Form for each young person you think may benefit from the program. The Referral Form requests information about the concerns you have for the young person as well as their strengths will be used as 'baseline data' from which to track participant progress in the program.

The Referrer must discuss their intent to refer a student to Yawardani Jan-ga with the parent/carer before completing a Referral Form and obtain parent/carer permission to share their contact details with Yawardani Jan-ga to begin the consent process.

Once Yawardani Jan-ga has received the Referral Form, we may contact you to clarify any information provided and provide an update on the enrolment of the young person referred.

Yawardani Jan-ga staff will 'check-in' with you mid-way (5-sessions), at the end of the program (all sessions completed), and six-months after the young person's last EAL-session (see Figure 1). These check-ins are expected to take between 5-10 minutes each.

The aim of checking in with the Referrer is to determine the participant's ability to apply knowledge, skills, and behaviours gained during Yawardani Jan-ga outside of the EAL-arena, to ensure the wellbeing of the student, and also help us adjust the program to the local context. The check-in at six months after the student's last EAL session will help us track the long-term impact of the EAL program on each young person, and find out if the student needs to re-engage with Yawardani Jan-ga.

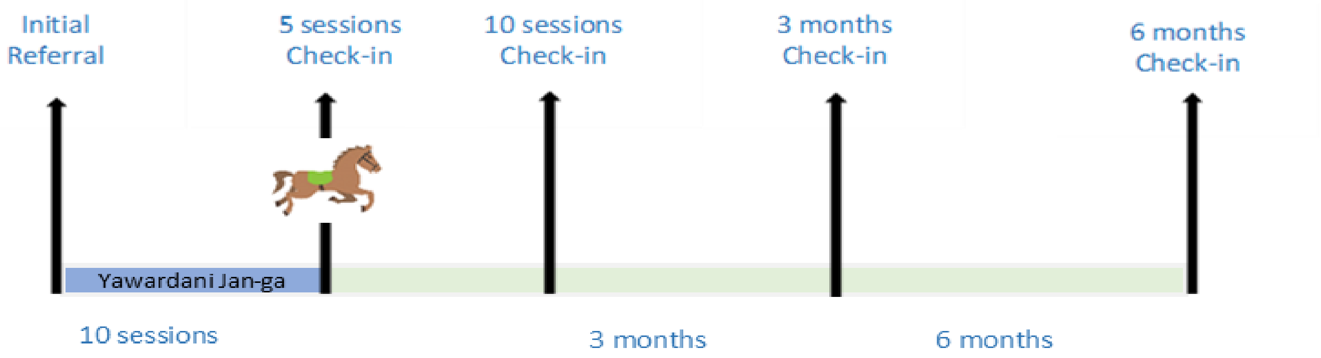

Figure 1: Timing of 'check-ins' with Referrers to track progress in Yawardani Jan-ga

What kind of information do you collect?

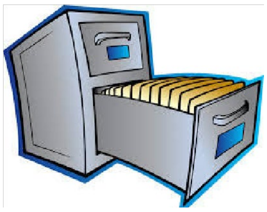

Yawardani Jan-ga will collect and store data pertaining to each participant referred to the program. This includes information such as the Referral Form, session notes, photos and videos of child-horse interactions, and feedback from check-ins with the Referrer.

These check-ins with the Referrers (and Parent/Carers) may be recorded using digital voice recorder or written notes, and will help us track the progress of each young person in the short and long-term and adjust sessions according to the student's needs. If we intend to record the check-in conversation, we will ask for your permission on the day.

At the end of the program, we will also ask the Referrer to check-in with the Yawardani Jan-ga team about their experience referring to the program. This information will help us evaluate and improve our processes to ensure the Yawardani Jan-ga is suitable to the context. At the end of each program, we will also collect feedback from EAL practitioners about what worked, what didn't and how the program and processes could be improved. In the future, with parent/carer consent, we will also be requesting school attendance data from public schools for students referred to Yawardani Jan-ga. This will help us understand if EAL helps to improve school attendance.

How will project information be kept private?

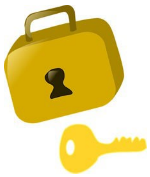

All information stored with Yawardani Jan-ga is confidential and is kept under lock and key at the EAL site and on a secure online app called JaneApp which can only be accessed via password protected computers by Professor Juli Coffin, the Program Manager, researchers and the EAL practitioners. EAL practitioners are only able to access information for the young people they individually support. Another secure online application, REDCap, is used to analyse the data. Both REDCap and JaneApp transfer and store data in encrypted form within Australia with access controlled by Professor Coffin.

Our team of researchers will review all information collected for each participant to independently and objectively assess the progress made by each young person from different perspectives. We will also use the combined information collected to evaluate the effectiveness of the program in improving social emotional wellbeing for different sub-groups of young Aboriginal people and to appropriately adapt the intervention program, referral and follow-up processes, training of EAL facilitators and evaluation tools to the Kimberley context.

The information we collect about each participant will not be shared with any other services without consent of the parents / carers and the participant themselves, unless:

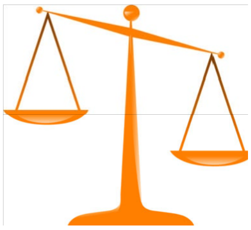

- the participant becomes involved in a serious criminal matter where others are at risk; or
- we perceive the participant to be at risk of self-harm or harm to others; or
- we are required to by the law.

In such instances, we will follow the required reporting and referral procedures, in consultation with each Principal.

Will the benefits of Yawardani Jan-ga be shared with the public?

Yes. General results about the whole program will be presented at community meetings and be published in the form of reports, newsletters, and journal articles. When general feedback about the program is given to community, no personal information about people involved will be made public. Any other presentations about the research findings or reports will not identify schools/organisations or individual participants involved.

What if I have a complaint?

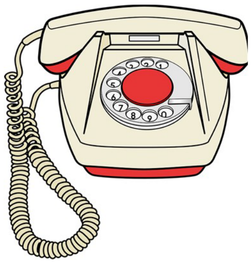

Approval to conduct this project has been provided by the Western Australian Aboriginal Health Ethics Committee (WAAHEC: HREC 926) and the Telethon Kids Institute's (TKI AEC 353) /Murdoch University Animal Ethics Committee. Any person with concerns or complaints about how we have behaved in your community may make any complaints by contacting the Western Australian Aboriginal Health Ethics Committee (08) 9227 1631 or by emailing ethics@ahcwa.org.

What if I have more questions or want more information?

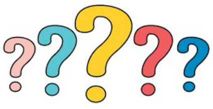

If you require further information or want to ask questions, please contact:

Professor Juli Coffin:

E: yawardani.smb@murdoch.edu.au
M: 0436 454 422

Our partners:

Yawardani Jan-ga
Information for School Principals

Welcome

Your school has been invited to participate in a research program for young Aboriginal people aged between 6 and 25. Ngangk Yira Institute are running the Yawardani Jan-ga Equine Assisted Learning (EAL) research program in multiple sites across the Kimberley including Broome, Derby, and Halls Creek. Through this research project we seek to understand what benefits EAL has for Aboriginal young people.

It is important that you read and understand the information below to decide if Yawardani Jan-ga may be suitable for some of your students.

What is the Yawardani Jan-ga research program about?

Yawardani Jan-ga means 'horses helping' in Yawuru language. The aim of the Yawardani Jan-ga project is to support the social, emotional, and spiritual wellbeing of Aboriginal young people through the implementation of culturally secure Aboriginal led and run EAL program.

In Yawardani Jan-ga horses are the teachers. Participants go through a series of interactions with horses under the supervision of a qualified local Kimberley Aboriginal EAL Practitioner. The interactions between participant and horse creates feedback loops which can support a young person to develop essential life skills including emotional regulation, self-awareness, socialisation skills and helpful self-thought. These life skills can help build healthy relationships with others, as well as demonstrate a different way of responding to life stresses.

Each participant attends weekly sessions for approximately one school term (10 weeks). Each session is normally around one hour in duration. Depending on the young person's referral, they may need to be enrolled longer or even on an on-going basis. Participants can attend for a variety of reasons and sometimes two students or more may attend together around thematic occurrences such as leadership, or where deemed appropriate. To ensure participation, for example two young people may attend together initially but then attend individual sessions. Sessions are held during school hours (8am – 2:15pm). In EAL the 'learning' component arises during activities with the horse that require skilled supervision, practice, and self-reflection. A mounted session may be offered; however, this is at the discretion of the local Aboriginal EAL Practitioner who supervises and guides all EAL activities during each session. To get to and from the EAL site, Yawardani Jan-ga program staff can often assist with providing transport for participants by arrangement.

What kind of participants are suitable for the program?

Young Aboriginal people between the ages of 6 and 25 are eligible to be part of the Yawardani Jan-ga program. The program is suitable for all Aboriginal young people, including those with complex social and emotional wellbeing needs, and those demonstrating leadership potential, the program provides prevention, intervention and treatment of issues facing young people. If a young person is receiving mental health

support, it is important they continue to receive mental health support during their enrolment in the Yawardani Jan-ga program.

What does a session look like?

Each session begins with a check-in around horse-safety and a meditation technique that tunes the participant to their environment and awareness of self. A range of topics are explored in Yawardani Jan-ga, meaning every session will be different and tailored to suit each young person how they present on the day. This planning is completed between the EAL Practitioner and lead researcher, Professor Juli Coffin, before and after every session.

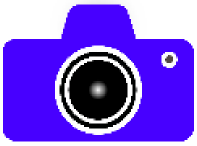

During the sessions, the EAL Practioner documents observations and things the child may have said or done. We will ask permission to take photos and or videos. These methods are some of the ways in which the program tracks the progress of each student during Yawardani Jan-ga, and also helps plan subsequent sessions.

Are there any risks to taking part in Yawardani Jan-ga?

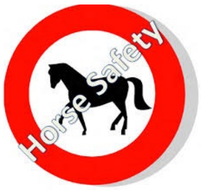

All participants are given a safety briefing every time there is contact with the horses. The horses are in a natural state and can move freely and quickly at times. The EAL Practitioner is responsible for monitoring the session and stopping and/or changing the experience if, in their opinion, there are any safety risks.

Should a young person become upset or need further support, the Yawardani Jan-ga program has support from local youth counselling services, and online / phone access to trained Aboriginal counsellors if an in-person visit is not possible. EAL does not deepen into emotional psychotherapy and the focus of EAL sessions remains on the young person's interaction with the horse and guided self-reflection.

How do students from my school access the program?

If an appropriate School Employee from your school is interested in referring a student to Yawardani Jan-ga, the following steps must occur (in order of appearance):

1. Principal Consent must be signed to allow school staff to refer students to the program.
2. School Employee must discuss their intent to refer a student to Yawardani Jan-ga with the parent/carer before completing a Referral Form and obtain parent/carer permission to share their contact details with Yawardani Jan-ga to begin the referral process.
3. School Employee must complete and return a Yawardani Jan-ga Referral Form and a Referrer Consent Form to **yawardani.smb@murdoch.edu.au** (a copy of the Referral Form and Referrer Consent is provided for your convenience). Please refer to the consent form.
4. Parents/carers must provide consent for all students under the age of 18 participating in Yawardani Jan-ga (A copy of the Parent/Carer Information Sheet and Consent Form is provided for your convenience).

It is important that the referrer discuss their intent to complete a Referral Form with the parent/carer and obtain parent/carer permission to share their contact information with Yawardani Jan-ga before completing a Referral Form.

A Yawardani Jan-ga employee will ensure that parent/carer consent is collected before a student is enrolled in the program. The Parent/Carer Consent Form includes a section asking for permission for the young

person to leave the school premises (or other location) during the school day to take part in Yawardani Jan-ga and permission for arrangements for getting to and from the program. Once parent and student consent has been provided, EAL sessions can be schedule for a suitable day and time for the student. If all Yawardani Jan-ga spots are full during a school term, the young person will be put on a wait list and the program will be offered as soon as possible.

Is a referral necessary?

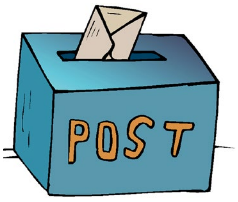

Yes. We require a completed Referral Form (copy attached) to be submitted to Yawardani Jan-ga for each individual student. The Referral Form requests information about the young person and will be used as 'baseline data' from which to track participant progress in the program.

Where a young person is receiving mental health support from the school, it is important that the young person continues to receive mental health support during their enrolment in the Yawardani Jan-ga program.

As a School Principal, what do I need to do?

As with any external educational program a school uses, it is the responsibility of each Principal to determine the program's educational suitability for each child or young person referred, and to ensure that it meets the requirements of the Department's Duty of Care in Public Schools Policy, the Excursions in Public Schools Procedures and the Recreation and Outdoor Education Activities for Public Schools Procedures. We will work with you to provide the evidence required to ensure these requirements are met.

The Yawardani Jan-ga program for students from [*insert name of school or location, e.g., Halls Creek*] will operate out of [*insert name of facility and location, e.g., for Halls Creek it will be Burks Park Station, 204 Duncan Highway, Halls Creek*].

As a School Principal, we request that your school help identify students that may benefit from EAL, and consent to appropriate School Employees working at your school to refer students to Yawardani Jan-ga and liaise with Yawardani Jan-ga staff to discuss student's progress. If you agree for your school to refer students to Yawardani Jan-ga, we will require each School Employee intending to refer students to sign a Referrer Consent Form (copy attached). Once we receive their Consent Form, they are able to commence referring students into the program by completing a Referral Form for each child they think may benefit from the program.

If you agree for your school to refer students, we also ask that you agree to the information collected during the referral process, during the operation of the program and at follow up points, be used to monitor student progress and adjust the program as well as for research purposes to evaluate the effectiveness of the program.

What kind of information do you collect?

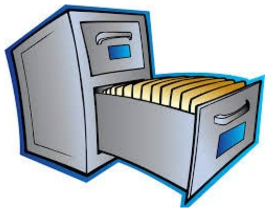

Yawardani Jan-ga will collect and store data pertaining to each participant referred to the program. This includes information such as the Referral Form, session notes, photos and videos of child-horse interactions, and feedback from check-ins with the Referrer.

Information collected from each Referrer will be limited to 'check-ins.' Yawardani Jan-ga staff will contact the Referrer after the student has completed 5 sessions,

Graduation (~10 sessions), and again in 3 months and 6 months after their Graduation from EAL. These check-ins will take about 5-10 minutes each time and can be done face-to-face or over the phone. The aim of checking in with the Referrer is to determine the participant's ability to apply knowledge, skills, and behaviours gained during Yawardani Jan-ga are being applied in the school setting and importantly to ensure the well-being of the student. The check-in at three and six months after the student's last EAL session will help us track the long-term impact of the EAL program on each young person, and find out if the student needs to re-engage with Yawardani Jan-ga. These check-ins may be recorded using digital voice recorder or written notes. Together, this information will help us track the progress of each young person in the short and long-term and adjust sessions according to the student's needs.

At the end of the program, we will also ask the Referrer to check-in with the Yawardani Jan-ga team about their experience referring to the program. This information will help us evaluate and improve our processes to ensure the Yawardani Jan-ga is suitable to the context.

We will also be doing similar check-ins with parents/carers and seeking feedback from EAL facilitators at the end of each program about what worked, what didn't and how the program and processes could be improved.

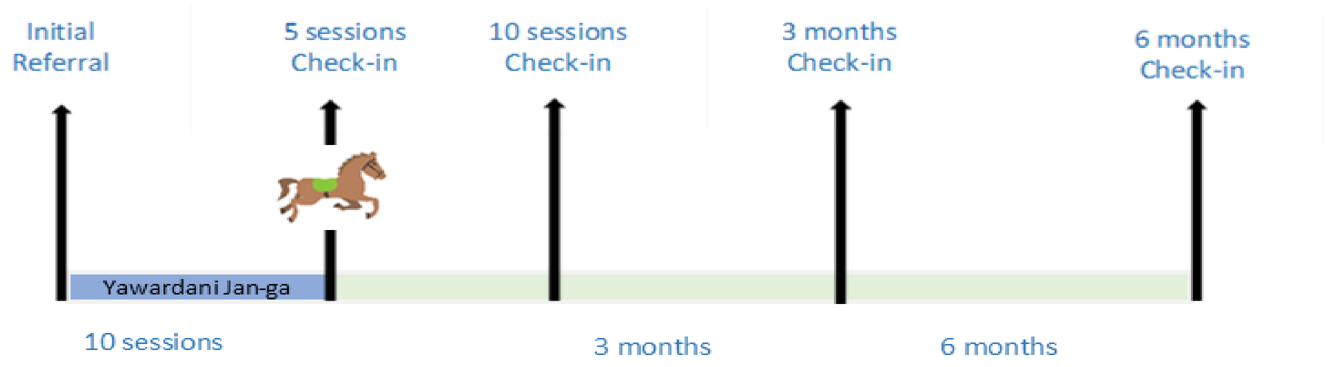

Figure 1: Timing of 'check-ins' with Referrers to track progress in Yawardani Jan-ga

In the future, with parent/carer consent, we will also be requesting 'school attendance' information for each student referred to Yawardani Jan-ga. In this instance, this means that for each student referred by WA Department of Education schools, information on attendance and absence rates (authorised and unauthorised absences) will be requested for the periods: a semester before, during and a semester after each young person has attended the program. We will also request, with your consent, aggregated school attendance data for comparison purposes (i.e., the average attendance and absentee rates for all students and all Aboriginal students in the same year levels as referred students. This overall attendance information will help us understand the association between school attendance and the EAL program for different groups of students (for example, gender, age, year level, reason for referral).

How will project information be kept private and how will it be used?

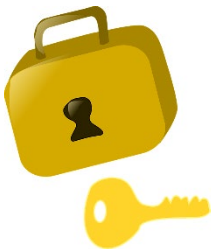

As employees of a research institution, all Yawardani Jan-ga employees sign agreements to treat all client information confidentiality. At the ground level, all information collected under this research program is treated confidentially and is stored under lock and key at the EAL Broome, Derby or Halls Creek sites and on password protected Murdoch University computers that only Professor Juli Coffin and the Program Manager have

access to. EAL practitioners are limited to access information for the young people they individually support.

During EAL sessions, the practitioners will use iPhones (not connected) to record photos and videos. These iPhones are password protected and processes are in place to ensure that photos and videos are offloaded from iPhones and stored on password secure iPad with storage provided through password protected and encrypted iCloud access. All images and video are downloaded after each session.

Our team of researchers will also have access to identified information collected about each student to confidentially and objectively assess the impact of EAL on each young person and determine if the skills gained in Yawardani Jan-ga are transferred into social settings. We will also use the combined information collected to evaluate the effectiveness of the program in improving social emotional wellbeing for different sub-groups of young Aboriginal people and to appropriately adapt the intervention program, referral and follow-up processes, training of EAL facilitators and evaluation tools to the Kimberley context.

The information collected about each participant will not be shared with any other services without consent from the parents/carers and the participant themselves, unless:

- the participant becomes involved in a serious criminal matter where others are at risk; or
- we perceive the participant to be at risk of self-harm or harm to others; or
- we are required to by the law.

In such instances, we will follow the required reporting and referral procedures, in consultation with each Principal.

Professor Juli Coffin is responsible for managing the monitoring plan and ensuring data is regularly collected and securely stored. All data collected about individual students, with informed parent consent, will be electronically stored within a secure hosted data base source called JaneApp which encrypts data in transfer and in storage on servers located in Sydney. Survey and referral data are collected and analysed using REDCap, a secure online survey platform, which also encrypts data in transit and storage, with data stored on secure servers located within Australia. Redcap only stores quantitative data and observations, it does not store photographic or video data of any sort, it is the higher analysis tool utilised for overall progress for the individual student and captured by numbers. Permission for program staff and researchers to access information stored on JaneApp or REDCap is controlled by CIA Professor Coffin, with confidential passcodes and access rights assigned by her. Highly confidential, paper-based information is locked in a stored cabinet in Professor Coffin's locked office. Following the Western Australian University Sector Disposal Authority's guidelines, any hard copy, electronic data, and digital recordings will be stored for a minimum of 7 years after publication or project completion or until students turn 25 years of age, whichever is later. After which time, electronic records will be destroyed via digital file shredding, and any hard copies of data will be destroyed through confidential shredding.

Do all members of the research team who will be having contact with children have their Working with Children Check?

Yes. All employees of the Murdoch University must have a Working with Children Check. This means all the non-research (administrative and EAL-Practitioners) and research staff (researchers) must undergo a Working with Children Check. In addition, as Yawardani Jan-ga offers transport to and from the EAL-site, our practitioner must also have a current valid driver’s license. Included in the attachments is a list of the current Yawardani Jan-ga team members who will be having contact with children from your school with their required documentation. Each staff member must also have a WA Police Clearance to obtain employment within Murdoch University and Yawardani Jan-ga.

Will the benefits of Yawardani Jan-ga be shared with the public?

General results about the whole program will be presented at community meetings and be published in the form of reports, newsletters, and journal articles. When general feedback about the program is given to community, no personal or identifiable information about individual people involved will be made public.

An annual summary of overall findings will also be provided to participating schools and the Department of Education.

Is this research approved?

Yes. This research has been approved by the Western Australian Aboriginal Health Ethics Committee (WAAHEC: HREC 926) and the Murdoch University Animal Ethics Committee (MU AEC 353). Various other organisations have provided their consent to refer children and young people to Yawardani Jan-ga.

As indicated in the attached letter, the ***research aspect*** of this project has approval from the WA Department of Education, on the condition that each Principal is satisfied that the intervention program is suitable for each child or young person referred from their school, and that the program, and the site at which it operates, meets the requirements of the Department's Duty of Care Policy, the Excursions in Public Schools Procedures and the Recreation and Outdoor Education Activities for Public Schools Procedures.

What if I have a complaint?

Any person with concerns or complaints about how we have behaved in your community may make any complaints by contacting the Western Australian Aboriginal Health Ethics Committee (08) 9227 1631 or by emailing ethics@ahcwa.org.

What if I have more questions or want more information?

If you require further information or want to ask questions, please contact:

Professor Juli Coffin

Mobile: **0436 454 422** Email: Yawardani.SMB@murdoch.edu.au

Our Partners:

The Kimberley Foundation

MINERAL RESOURCES

MARY MACKILLOP today

PROUDLY SUPPORTED BY telethon 7

Australian Government National Health and Medical Research Council

NHMRC

healthway

PRIVATE-CONFIDENTIAL

Yawardani Jan-ga
Principal Consent Form

I ____ have read the "Information for School Principals" and:
(Principal name)

I understand the aims, procedures, and risks of this project, as described within it.	Yes ☐ No ☐
I understand that participation in Yawardani Jan-ga research project is entirely voluntarily.	Yes ☐ No ☐
I understand I am responsible for ensuring the program is suitable for each referred student, and that it meets the requirements of the WA Department of Education's Duty of Care Policy and Excursions in Public Schools Policy and Procedures.	Yes ☐ No ☐
I understand Yawardani Jan-ga staff will discuss with me how students will get to and from the program and ensure parents/carers have given informed consent for the agreed arrangements	Yes ☐ No ☐
I am willing for my school to become involved in the research project.	Yes ☐ No ☐
For any questions I may have had, I have taken up the invitation to ask those questions, and I am satisfied with the answers I received.	Yes ☐ No ☐
I understand that employees (e.g. teachers, psychologists, teacher assistants) working at my school will be requested to complete, with parent/carer consent, a Yawardani Referral Form about children they think may benefit from the program.	Yes ☐ No ☐
I understand that students that become enrolled in Yawardani Jan-ga must leave the school premises for a minimum for one hour per week.	Yes ☐ No ☐
I understand that Referrers (e.g. teachers, psychologists, teacher assistants) will be contacted by Yawardani Jan-ga staff to 'check-in' about the students they have referred to the EAL-intervention, as well as provide feedback about their experience with referring to the program.	Yes ☐ No ☐
If a student enrolled is requested to complete a Media Consent Form, the Principal of the school must be advised and notified prior to photographs or video being used for media purposes. Parent/Carer consent will also be required.	Yes ☐ No ☐
I understand that all information provided by my staff will be combined with information collected from others, to monitor the progress of each child as well as evaluate the overall effectiveness of the program and to appropriately adapt the intervention program, referral and follow-up processes, training of EAL facilitators and evaluation tools to the Kimberley context.	Yes ☐ No ☐
I understand that my school will be provided with a copy of the findings from this research upon its completion.	Yes ☐ No ☐
I understand that my school is free to withdraw its participation at any time. However, it may not be possible to withdraw all data pertaining to the school from the study results if data has already been de-identified for evaluation and research purposes.	Yes ☐ No ☐
I understand that this research may be presented in a variety of forms, including at community and scientific meetings and forums, as well as conferences, published in reports, and in journals, provided that the student participants, staff or the school are not identified in any way.	Yes ☐ No ☐

Name of Principal & Signature:

____

School Name:

____Date______________________

PRIVATE-CONFIDENTIAL

Yawardani Jan-ga
Participant Information Sheet

Welcome

The Ngangk Yira Institute for Change, Murdoch University is running the Yawardani Jan-ga (Horses doing healing) Equine Assisted Learning (EAL) research program in Broome, Derby, Halls Creek, and other localities in the Kimberley region.

You are invited to be part of the program. In the program you will learn important skills from the horses, for example how to communicate well with others by reading your own body language as well as others. These skills can help us build healthy relationships with others, and help us handle life stresses better.

It is important to know about Yawardani Jan-ga and what it involves before saying "Yes" or "No" to taking part.

What is the Yawardani Jan-ga research program about?

In this research, we are seeing what benefits EAL has for young people.

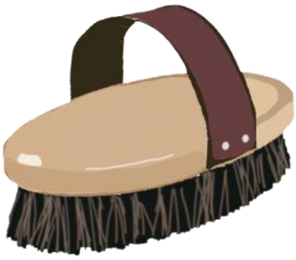

In Yawardani Jan-ga horses are the teachers. During each session you will work with, or around horses on the ground doing activities with the horse, following instructions from an EAL Practitioner. Activities change according to each session and can include grooming observing or grooming / washing a horse.
A mounted session may be offered towards the end of the program, if the EAL Practitioner thinks it is safe for you to do.

What do I have to do?

For Yawardani Jan-ga, you do not need any experience with horses.

The program goes for a session a week for about one school term (10 weeks), but you may be enrolled longer or even on an on-going basis if you need to.

Each session lasts 45 minutes to 1-hour and are held during school hours (8am – 2:30pm).
Yawardani Jan-ga program staff will discuss with your parents/carers and the school Principal to agree about how you will get to and from the program either from home or school.

Each session begins with a horse safety check followed by a check-in around your wellbeing.

Each session will be different. The focus is on the experience that you have with and the feedback you get from the horse, which will be guided by a trained EAL Practitioner.

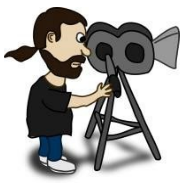

During the sessions, the EAL Practitioner might take notes and make plans for the next session. Sometimes, **photos**, **videos**, or **voice recordings** will be taken of what you are learning or experiencing during the sessions.

Each session, you can say 'yes' or 'no' to having a photo taken, or a voice or video recorded. It will not stop you child from being in Yawardani Jan-ga.

6-months after the last EAL session, we will check-in again to see how you are going. We will also ask for permission to contact you in the future. This will help us see how long the effects of EAL last.

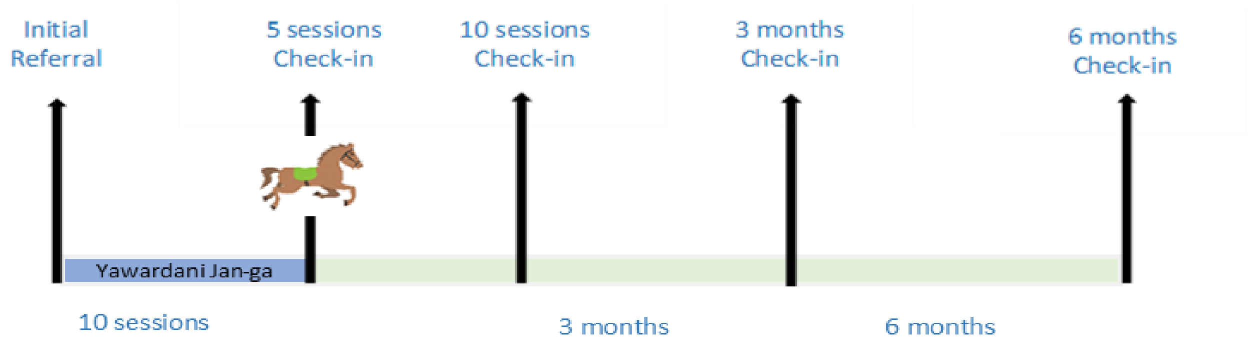

Figure 1: Timing of 'check-ins' with Referrers to track progress in Yawardani Jan-ga

What if I don't want to do one of the activities in the session?

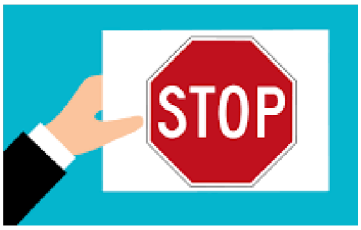

If we start a session and you decide that you want to stop, you can stop the session by saying to your EAL Practitioner that you want to stop.

In the Yawardani Jan-ga program, you are in charge and can say 'yes' or 'no' to conversations and activities. Please remember that participation is a choice, and up to you. No one will mind if you want to stop.

Will taking part in this Program upset me?

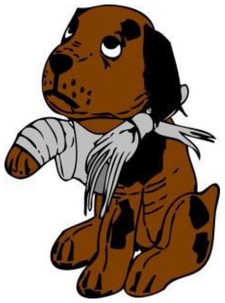

You will be working with and around horses. Horses are big animals and can sometimes act in sudden and unpredictable ways, especially if frightened or hurt. The horses are in a natural state and can move freely and quickly at times.

You will be given safety instructions every session. You will need to follow our instructions at all times.

The EAL Practitioner is responsible for keeping a close eye on how you behave around the horses and can stop and/or change the experience if, in their opinion, there are any risks to your safety.

If you become upset or need support, the Yawardani Jan-ga program has support from local youth counselling services, and online/phone access to trained Aboriginal counsellors if a face-to-face visit is not possible.

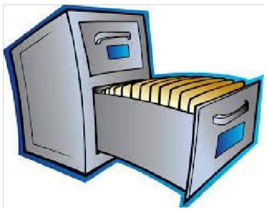

Will you collect personal information?

Yes. Yawardani Jan-ga will keep a 'Participant-file' with all your information in it. It will contain information about you, such as the notes, photos, videos, that your EAL practitioner makes during each session and other feedback about how you are going from your teachers, parents/carers, and other people close to you. We will also be checking in with you 6-months after the program to find out what you thought about it.

We will also check-in with the organisation that referred you to us about how you are doing. This will help us understand what you are learning from the horses.

We also want to combine the information we collect about you with information we collect about other young people coming to Yawardani Jan-ga. This will help us work out if and how the program helps Aboriginal young people as a group and helps us make the program better for other Kimberley communities.

With your permission and the permission of your parent/carer, we will also ask your school to provide information about how often you attend school in the semester before you started with Yawardani Jan-ga, while you are at the program and the semester after you have finished.

How will you keep my information private?

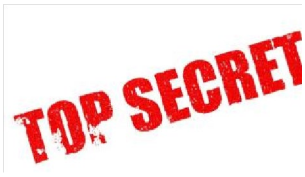

All information stored with Yawardani Jan-ga is **confidential** and is kept under lock and key at the EAL site and on a password protected computer that only the Yawardani team has access to.

Our team of researchers will review all the information we have collected about you independently and confidentially. This means that your name will not be shared with anyone outside of the Yawardani Jan-ga team, **unless**:

- you become involved in a serious criminal matter where others are at risk; or
- we think you might be at risk of harming yourself or harming others; or
- we are required to by the law.

What will you do with the information you collect?

The information collected by the EAL practitioners, such as notes, photos, video, voice recordings and check-ins with parents, teachers and others close to you, will be used to understand the benefits the horses are having on you, and other young people enrolled in the Yawardani Jan-ga program. We will also use what we find out to improve the program for future young people.

We would also like to ask your permission to contact you **in the future** to see how you are going. For example, we might ring you in a year's time to ask what you are up to. Right now, we are only asking for permission to contact you. When we contact you in the future, you can say 'yes' or 'no' to speaking to us. It will be completely up to you.

We will keep your information safe until you turn 25 years of age and then it will be destroyed.

Do I have to say yes?

No – not at all. It's up to you! Just say if you don't want to join in. Nobody will mind. If you say yes at first and then decide you don't want to join Yawardani Jan-ga, that's ok as well.

What should I do now?

Now you know about the study, you need to think if you want to take part in the study and talk to your carers about it.

What if I want more information?

You can contact Juli (Program Coordinator) on phone, text, or email: 0436 454 422 or yawardani.smb@murdoch.edu.au ☺

Our partners:

The Kimberley Foundation

MINERAL RESOURCES

MARY MACKILLOP today

PROUDLY SUPPORTED BY telethon 7

Australian Government National Health and Medical Research Council

NHMRC

healthway

PRIVATE-CONFIDENTIAL

Yawardani Jan-ga

Participant Assent Form (6-15 years)

To be filled-in by the child and their parent/caregiver

PLEASE CIRCLE ALL YOU AGREE WITH:

Has somebody explained Yawardani Jan-ga to you?	Yes ☐	No ☐
Do you agree for photos or video of you and horses to be taken?	Yes ☐	No ☐
Do you agree to the check-ins with Yawardani Jan-ga staff about your progress?	Yes ☐	No ☐
Do you agree for the school to give us your attendance information?	Yes ☐	No ☐
Do you understand it's OK to say 'stop' or say 'no' at any time?	Yes ☐	No ☐
Are you happy to take part?	Yes ☐	No ☐

IF you want to take part, please write your name and today's date

Your Name: ____

Today's date: ____

Nov 2023

PRIVATE-CONFIDENTIAL

Yawardani Jan-ga

Participant Consent Form (16+)

By signing this consent form I agree to participate and show that I have understood the following:

- I understand what this project is trying to find out what benefits EAL has on our mob, and how to make the program better.
- I understand the risks of taking part in this project.
- That I am taking part because it is my choice, and I can say ‘no’ or ‘stop’ at any time.
- I understand that the purpose of ‘check-ins’ between myself and Yawardani Jan-ga staff are to find out how the program is helping me.
- I understand that Yawardani Jan-ga staff will check-in with the organisation that referred me to the program to track my progress.
- I understand that Yawardani Jan-ga will ask my school to provide information about my attendance to school to see if the program changes my attendance.
- I understand that my information will be combined with information collected from all other young people coming to Yawardani Jan-ga to see if and how the program helps our mob, and how to make the program better for others.
- I understand that information about me will be stored confidentially and will only be shared to ensure my safety.
- That general feedback about the program given to community will not reveal my name or personal information.
- I have been given enough information and the opportunity to ask questions. I have been given a copy of the Information Sheet to take home.

I ____ have read and understood the "Participant
(young person's name)

Information Sheet" and agree:

To participate in the Yawardani Jan-ga Program.	Yes ☐ No ☐
For photographs, video footage and voice recordings to be taken during the program	Yes ☐ No ☐
For my school to give Yawardani Jan-ga information about my school attendance.	Yes ☐ No ☐
For all information Yawardani Jan-ga collects about me to be used to find out if the program is working for me, for our mob, and how to make the program better for others.	Yes ☐ No ☐
I give permission to be contacted in the future.	Yes ☐ No ☐

Young person's signature: ______________________________ Today's date: _______________________

Thank you for taking part in Yawardani Jan-ga.

PRIVATE-CONFIDENTIAL

Yawardani Jan-ga
Participant Check list (18+)

Date completed:	
name of person(s) completing check list:	

CONTACT INFORMATION

Name of young person:		*Weight (kg):*
Who should we contact in case of an emergency (name & phone number):		relationship to you?
Alternate contact person name and phone number:		relationship to you?

HEALTH HISTORY

Please indicate any medical condition that may make it impact participation in this program Yawardani Jan-ga

	**Details / Management (e.g. medication, therapy)**
Do you have any allergies:	Tick all that apply:<br>☐ To animal hair *(details):*<br>☐ To any medicines (details):<br>☐ Asthma *(details):*<br>☐ Other *(details):*
Are you taking any medications: *in the case on an emergency, incident or injury, this information will help us inform first aid responders.*	Please tick:<br>☐ No<br>☐ Yes (*details):*
Is there any other information you think we should know you?	

Nov_2023
